# Single‐Cell Transcriptomics Reveal Microenvironment Alterations in Canine Peri‐Implantitis

**DOI:** 10.1155/mi/9937505

**Published:** 2025-12-31

**Authors:** Ming Wang, Dong Zhang, Chunhui Liao, Daner Wu, Ningbo Geng, Yixin Xia, Yu Chen, Songling Chen, Wei Peng

**Affiliations:** ^1^ Department of Stomatology, The First Affiliated Hospital of Sun Yat-sen University, Guangzhou, 510080, Guangdong Province, China, sysu.edu.cn; ^2^ Department of Oncology, The First Affiliated Hospital Sun Yat-sen University, Guangzhou, 510080, Guangdong Province, China, sysu.edu.cn; ^3^ Department of Orthodontics, Guangzhou Women and Children’s Medical Center, Guangzhou Medical University, Guangzhou, 510080, Guangdong Province, China, gzhmc.edu.cn

**Keywords:** canine disease model, peri-implantitis, single-cell RNA sequencing, tissue-specific microenvironment

## Abstract

**Background:**

Peri‐implantitis, a major contributor to dental implant failure, lacks comprehensive insights into tissue‐specific heterogeneity as current researches predominantly focus on the whole peri‐implant tissue rather than distinct molecular and cellular dynamics in gingiva and alveolar bone microenvironments. Furthermore, ethical challenges hinder the acquisition of healthy peri‐implant tissues, limiting our understanding of peri‐implantitis progression and the development of targeted therapies.

**Methods:**

We established a controlled peri‐implantitis model in beagle dogs, enabling ethical collection of healthy control tissues. Single‐cell RNA sequencing (scRNA‐seq) transcriptomics profiling was conducted on gingiva and alveolar bone tissues from diseased and healthy controls. Additionally, flow cytometry was utilized to further verify the identified subclusters and their involvement in peri‐implantitis.

**Results:**

Single‐cell transcriptomic profiling unveiled a pronounced expansion of inflammation‐associated cellular subsets in both gingival and alveolar bone microenvironments during peri‐implantitis. Gingival tissues exhibited marked expansions in IL6^+^/IL18BP^+^ endothelial cell and CXCL8^+^ fibroblast, whereas APOD^+^ fibroblast dominated in peri‐implantitis bone tissues. Gene‐level profiling further identified upregulated pro‐inflammatory chemokines (*CXCL8*, *CXCL17*, *CCL24*) within gingiva IL18BP^+^ endothelial cells. Notably, we discovered a unique ligand–receptor interaction C3 (APOD^+^ fibroblast)–C3AR1 (monocyte/macrophage) in alveolar bone tissue, implicating complement‐dependent signaling in immune crosstalk.

**Conclusions:**

Our study provides the first comparative atlas of soft/hard tissue remodeling in peri‐implantitis at single‐cell resolution. The expansion of IL6^+^/IL18BP^+^ endothelial cell and CXCL8^+^ fibroblast in gingiva, alongside APOD^+^ fibroblast‐driven C3–C3AR1 signaling in alveolar bone, highlights distinct microenvironmental reprogramming between soft and hard tissues. These findings not only identify potential therapeutic targets but also validate the translational relevance of the canine model for peri‐implantitis research.

## 1. Introduction

Peri‐implantitis is a common complication characterized by chronic inflammation of the peri‐implant tissue. This disease features irreversible and progressive degradation of gingival and alveolar bone, ultimately leading to tooth loss [1, 2]. A systematic review reported a weighted mean prevalence of 22%, highlighting peri‐implantitis as the leading cause of tooth‐implant surgery failure [2]. However, on the basis of the current understanding, the treatment for this disease remains limited to removing the biofilm, which usually results in unsatisfactory outcomes [3–5]. A better understanding of the underlying cellular and molecular dynamics of the gingiva and alveolar bone during peri‐implantitis has broad implications for both biological understanding and therapeutic targeting of the pathological peri‐implantitis.

In order to overcome the challenges, studies have highlighted the significant roles of both immune and nonimmune cells in tissue destruction [6, 7]. T cells are crucial for disease progression, and the present studies have revealed increased expression of *RORγT* and *FOXP3* in affected tissues [8], indicating a role for TH17 and Treg cells in peri‐implantitis. Additionally, nonimmune cells such as fibroblasts have been shown to promote inflammation in peri‐implantitis through interactions with neutrophils [9]. Moreover, endothelial cells can regulate angiogenesis and vasculogenesis via vascular endothelial growth factors (VEGFs) to influence immune cell migration [10]. However, most current studies were based on entire peri‐implant tissue, limiting a thorough understanding of the heterogeneity of gingival and alveolar bone tissues as well as the development of targeted host therapy for peri‐implantitis.

To date, the pathogenic molecular and cellular mechanisms involved in peri‐implantitis remain incompletely understood. The advent of single‐cell RNA sequencing (scRNA‐seq) has provided insight into the transcriptome of individual cell in tissues [7]. However, current researches predominantly focus on the whole peri‐implant tissue rather than distinct molecular and cellular dynamics in the gingiva and alveolar bone microenvironments separately. Furthermore, ethical challenges hinder the acquisition of healthy peri‐implant tissues, limiting our understanding of peri‐implantitis progression and the development of targeted therapies. Herein, we utilized single‐cell RNA sequencing to investigate inflammation‐associated cells in gingival and alveolar bone tissues separately obtained from beagle dogs. Additionally, all protocols of our study complied with the Animal Research: Reporting of In Vivo Experiments (ARRIVE) guidelines [11].

## 2. Materials and Methods

### 2.1. Animals

The study involved four healthy, 1‐year‐old male beagle dogs. The surgical procedures involved bilateral extraction of the mandibular second, third, and fourth premolars simultaneously under general anesthesia. Following a 12‐week healing period, 24 dental implants (φ3.6 mm, H7 mm, Dentium, Seoul, South Korea) were placed into the edentulous sites. Each implant had a placement torque of at least 35 N·cm. After 1 week, cover screws (φ3.4 mm, H0 mm, Dentium, South Korea) were attached to the implants. Abutments (φ4.5 mm, H3.5 mm, Dentium, South Korea) were connected 8 weeks postimplantation [12]. All methods and protocols were authorized by the Institutional Animal Care and Use Committee at Sun Yat‐sen University (Approval No. SYSU‐IACUC‐2022‐‐000486).

### 2.2. Induction of Peri‐Implantitis and Sample Collection

After installation of the healing abutment, 3–0 silk ligatures were placed below the healing abutment of the left‐side implants of each beagle dog to induce inflammation through plaque accumulation, whereas the right side did not receive ligatures. The control side underwent daily oral hygiene with 0.12% chlorhexidine gluconate. Ligature retention and implant stability were manually assessed every 48 h. After an 8‐week observation period, the animals were sacrificed via intracardiac injection of 10% potassium chloride (0.5 mL/kg). Mandibles were surgically excised, and gingival tissues and alveolar bone samples were collected separately for downstream analyses. One mandible was collected for micro‐CT analysis to confirm the success of the model construction. The other mandibles were used to obtain the peri‐implant tissues including gingival and alveolar bone tissues from the location of third and fourth premolars of canines for single‐cell suspensions and the remaining peri‐implants tissues were harvested for the further flow cytometry. Peri‐implantitis samples were selected based on clinically evident inflammation (bleeding on probing positive, probing depth >5 mm). Gingival tissues were harvested by elevating full‐thickness flaps and collecting 4 mm‐wide circumferential biopsies from the implant–gingiva interface. Alveolar bone tissues were obtained from a cylindrical area with a 1‐mm radius around the implant surface and extending 4 mm in length from the top of the bone level with a caries scoop following complete removal of the gingival.

### 2.3. Computed Tomography Analysis

Micro Computed Tomography (Inveon, Siemens, Germany; 80 kV, 500 μA, 1500 ms exposure time) was used to image the surrounding tissues of the implants in both sides. alveolar bone loss was quantified via three‐dimensional reconstruction. The alveolar bone height was measured in the lingual and palatal regions, and the four regions measured yielded an average amount of bone loss [12].

### 2.4. Single‐Cell RNA Sequencing and Analysis

#### 2.4.1. Sample Preparation

To prepare single‐cell suspensions, alveolar bone was cut into small pieces (<1 mm^3^), and gingival tissue was minced and digested separately with collagenase type I (Gibco) and dispase II (Sigma) as previously described [13]. Red blood cells were subjected to lysis using 1 mL of Red Cell Lysis Solution (Biosharp, China). The cells were subsequently centrifuged and resuspended in 1% BSA solution. Following resuspension, the cells were passed through a 40‐μm filter (Biosharp), washed, and counted with a Countess 3 Automatic cell counter (Thermo Fisher, USA).

#### 2.4.2. 10 × Genomics Library Construction

Single‐cell RNA sequencing libraries were prepared via the Chromium Single‐Cell 3ʹ Reagent Kit v3 (10× Genomics) according to the manufacturer’s instructions. Briefly, ~35,000 cells/FACS‐sorted cells were washed and resuspended to a concentration of 700–1200 cells/µL (viability ≥ 85%) as described in a previous study [14]. After the reverse transcription step, barcoded cDNA was purified with Dynabeads, followed by PCR amplification. For gene expression library construction, 50 ng of amplified cDNA was fragmented and end‐repaired, double‐sized with SPRIselect beads, and sequenced on a NovaSeq platform (Illumina) to generate 150 bp paired‐end reads.

#### 2.4.3. Mapping and QC Filtering

Raw sequencing reads were first demultiplexed and mapped to the reference genome using Cell Ranger (v3.0.0). Subsequent analyses were performed in R (v4.2.2) with the Seurat package (v4.3.0). For quality control, cells with >20% mitochondrial gene expression were discarded. We also excluded cells with insufficient transcript detection (<350 genes for healthy samples or <250 genes for diseased samples) as well as those with unusually high gene counts (>6500 for healthy and periodontitis samples or >6000 for peri‐implantitis) as described in a previous study [9].

#### 2.4.4. Dimension Reduction and Unsupervised Clustering

To mitigate the effects of cell cycle heterogeneity on cell clustering, G2M and S phase scores were calculated via the function CellCycleScoring [15]. G2M and S phase scores were used to “regress out” heterogeneity due to the cell cycle via ScaleData. We conducted the principal component analysis (PCA) on highly variable genes (HVGs) to reduce the dimensionality of the data. The top 40 principal components were selected for unsupervised clustering of single cells. Nonlinear dimensional reduction (uniform manifold approximation and projection [UMAP]) was then used to visualize the clustering results (resolution = 0.6).

#### 2.4.5. Differential Gene Expression Analysis

To identify differentially expressed genes (DEGs) in each cluster, FindAllMarkers with the Wilcoxon rank‐sum test algorithm and Benjamini–Hochberg correction were performed to reduce the bias caused by multiple tests with the following thresholds: |log_2_ (fold change) | >2.0 and adjusted *p* value <0.05. The following configurations were set as previous study [9]. We used the EnhancedVolcano R package to visualize the DEGs between peri‐implantitis canines and healthy controls.

#### 2.4.6. Enriching Pathway Analysis

GO and KEGG enrichment analyses of the DEG sets were performed using the GO‐seq R and KOBAS packages, respectively. GO and KEGG terms with adjusted *p* values < 0.05 were considered statistically significant.

#### 2.4.7. Cell Communication Analysis

We applied via the CellPhoneDB, a public repository of ligands and receptors and their interactions. Membrane‐secreted and peripheral proteins of the cluster at different time points are annotated. Significant interaction means and cell communication significance (adjusted *p* value < 0.05) were calculated on the basis of the interaction and the normalized cell matrix achieved by Seurat normalization.

### 2.5. Flow Cytometry

Single‐cell suspensions were washed twice with the ice‐cold flow cytometry (FACS) buffer (2% FBS + 1 mM EDTA in PBS) and incubated with blocking buffer (1:100, 564765, BD Bioscience) for 15 min at 4°C. For cell surface antigen staining, single‐cell suspensions were stained with CD45 (553081, BD Bioscience) and CD11b (101236, BioLegend) in the dark for 1 h on ice and then washed two to three times with FACS buffer. For intracellular cytokine detection, the cells were fixed and permeabilized with fixation/permeabilization buffer (BD Bioscience) according to the manufacturer’s protocol. Cells were then stained with CD3 (553066, BD Bioscience), B220 (561881, BD Biosciences), and Ly6G (551461, BD Bioscience) antibodies. Data analysis was performed by using FlowJo software (Tree Star, Ashland, OR, USA).

### 2.6. Statistical Analysis

Data analysis and visualization were performed with Prism (GraphPad). The *p* values were calculated using the unpaired two‐tailed student’s *t* test or one‐way ANOVA followed by Tukey–Kramer multiple‐comparisons test. *p* values < 0.05 were considered statistically significant and are displayed as follows: ns, no significant, *p*  > 0.05:  ^∗^ *p*  < 0.05;  ^∗∗^ *p*  < 0.01:  ^∗∗∗^ *p*  < 0.001 and  ^∗∗∗∗^ *p*  < 0.0001. Data were expressed as mean ± SEM.

## 3. Results

### 3.1. Single‐Cell RNA Sequencing Performed Separately on Gingival and Alveolar Bone Tissues From Both Healthy Samples and Those With Peri‐Implantitis

To observe the heterogeneity between alveolar bone and gingival tissues under peri‐implantitis versus healthy samples, we performed scRNA‐seq on these tissues (Figure 1a). After standard data processing and quality filtering (Supporting Information 1: Figure S1a), we obtained single‐cell transcriptomes from a total of 35,039 single cells. UMAP clustering identified 13 distinct cellular compartments, annotated through canonical marker gene expression (Figure 1b,d and Supporting Information 1: Figure S1b).

Figure 1Overview of the 35,039 single cells from bone and gingiva tissues in peri‐implantitis and healthy control separately. (a) Study overview: cell suspension was prepared from peri‐implantitis groups (imflamed) and healthy controls (healthy) of beagle dogs. The picture shows the position of the biopsies we obtained. Next, we performed the single‐cell RNA sequencing and verified by flow cytometry. (b) Uniform Manifold Approximation and Projection (UMAP) of the 35039 cells, colored by cell‐type annotation from left to right: healthy bone (HB), peri‐implantitis bone (PB), healthy gingiva (HG) peri‐implantitis gingiva (PG). Epi, epithelial cells; Endo, endothelial cells; Fib, fibroblast cells; Neu, neutrophils; LEC, lymphatic endothelial cells; Mon/mac, monocytes/macrophages; SMC, smooth muscle cells. (c) Proportion of each cluster of HB, PB, HG, PG. (d) Dot plot depicting the expression of cluster‐defining genes and percentage of cells expressing each gene for the 13 clusters, respectively. Expression values are normalized and scaled averages. avg.exp, average expression; %.exp, percentage expression. (e) The upper panels depict a representative flow‐cytometry scatterplots of alveolar bone tissue, while the lower depict the flow‐cytometry scatterplots of the gingival tissue. Cells were gated from single/live and stained with CD45 (*n* = 3/group, error bars represent SEM,  ^∗^ *p*  < 0.05 as determined by unpaired two‐tailed Student’s *t* test).

**Figure:**
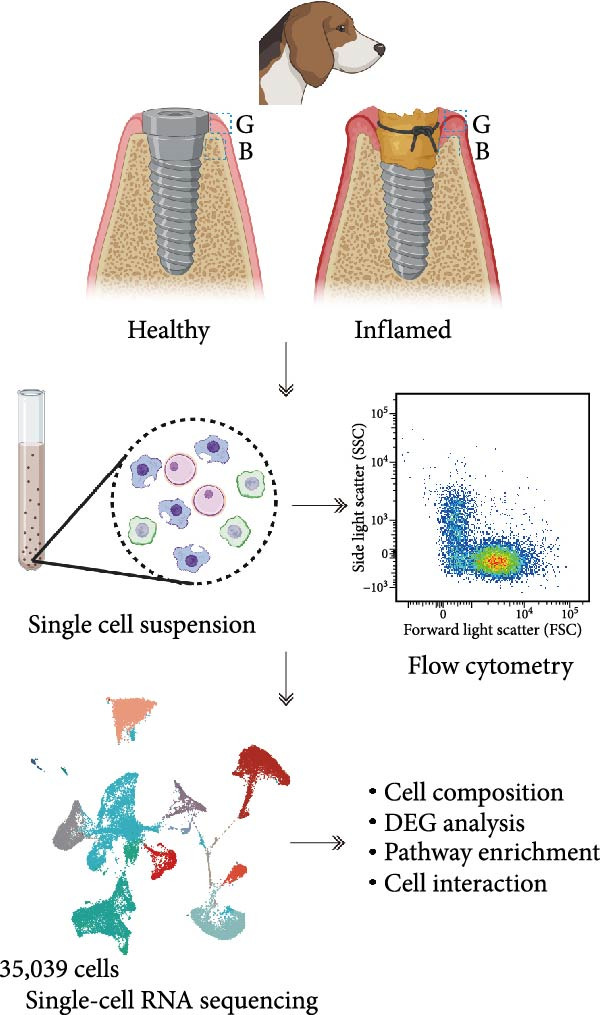
(a)

**Figure:**
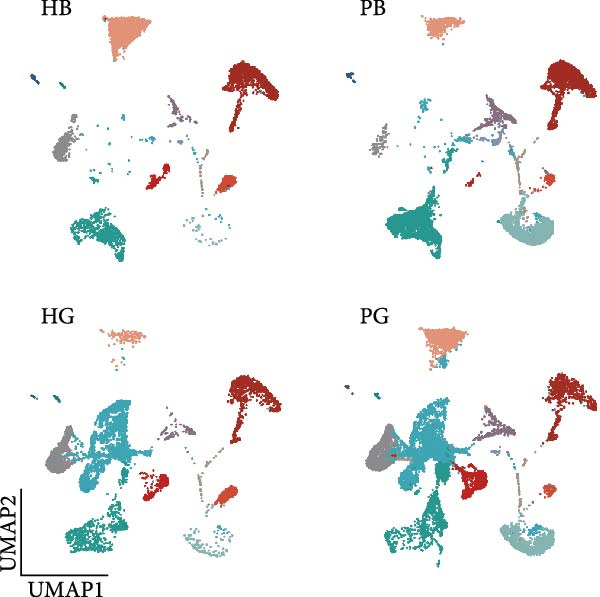
(b)

**Figure:**
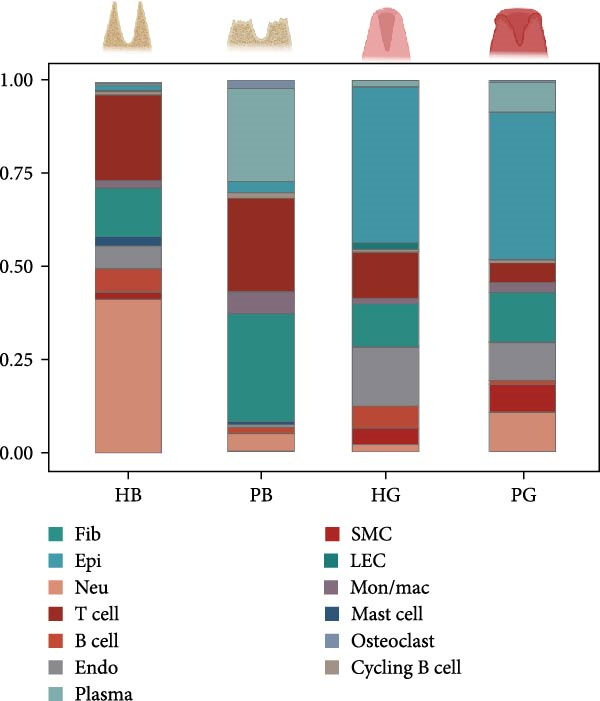
(c)

**Figure:**
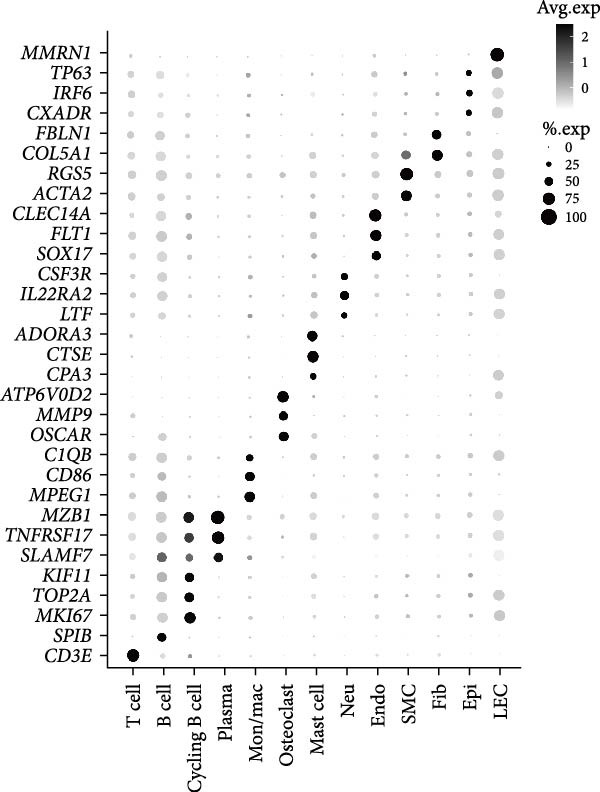
(d)

**Figure:**
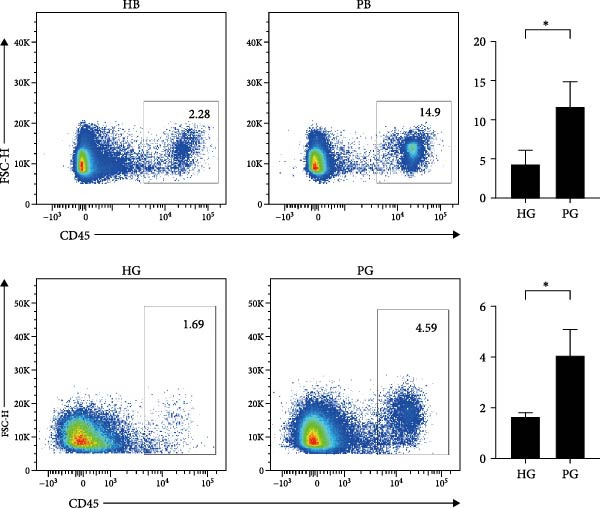
(e)

We next compared the proportion and quantity across tissue types (Figure 1c and Supporting Information 1: Figure S1c), revealing part of parallel inflammatory dynamics in both tissues during peri‐implantitis. Immune cells, such as plasma cells, monocyte/macrophage (Mon/Mac) and cycling B cell were significantly increased in both peri‐implantitis bone (PB) and peri‐implantitis gingival (PG) tissues, with plasma cell showing the most substantial increase. Conversely, B cell and lymphatic endothelial cell (LEC) decreased in the PB and PG, consistent with previously reported [7, 16]. As for nonimmune cells, we observed an increase of fibroblast (Fib) in both tissues. In contrast, the proportions of neutrophil (Neu), mast cell and endothelial cell (Endo) decreased in alveolar bone tissues but increased in gingiva, whereas the proportions of T cell increased in alveolar bone tissue but decreased in gingival tissue (Supporting Information 1: Figure S1d).

In general, both tissue types exhibited severe inflammatory responses verified by flow cytometry (Figure 1e), whereas the immune activation patterns diverged significantly between bone and gingival microenvironments. Alveolar bone tissues presented a more pronounced increase in nonimmune cells, particularly fibroblast, whereas gingival tissue exhibited a more pronounced increase in immune cells, particularly neutrophil and plasma cell (Supporting Information 1: Figure S1e).

### 3.2. The Diverse Functions of Fibroblasts Have Been Revealed and Are Particularly Associated With Inflammation

Given the dramatic expansion of fibroblast in both tissues under inflammatory conditions, we next focused on the functional heterogeneity of the subclusters (Figure 2a).

Figure 2Distinct subclusters of the fibroblast. (a) UMAP of fibroblast cells as in Figure 1a, annotated and colored by the sample type of origin (HB, PB, HG, PG) and subclustering. (b) Proportion of each cluster in HB, PB, HG, PG. (c) Dot plot depicting the expression of cluster‐defining genes and percentage of cells expressing each gene for the five subclusters of fibroblast respectively. Expression values are normalized and scaled averages. (d) Gene enrichment analysis of CXCL8^+^ Fib associated with genes upregulated between HG versus PG. (e) Gene enrichment analysis of APOD^+^ Fib associated with genes upregulated between HB versus PB. (f) Gene enrichment analysis of SFN^+^ Fib associated with genes upregulated between HB + HG versus PB + PG. (g) Volcano plot depicting the increased expression of *SFN* and *GPX2* in PG. Red points represent the genes with FDR < 0.1, log_2_ (fold change) > 2.0 and adjusted *p* value < 0.05 in PG.

**Figure:**
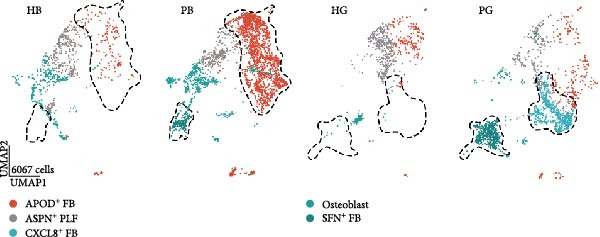
(a)

**Figure:**
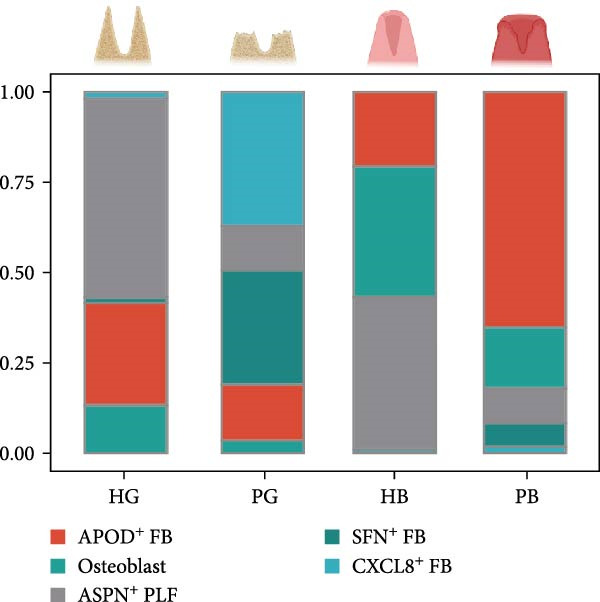
(b)

**Figure:**
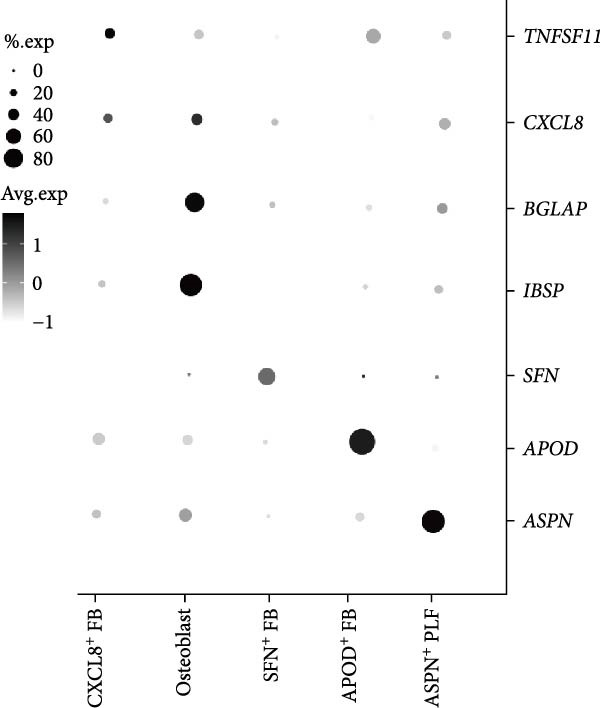
(c)

**Figure:**
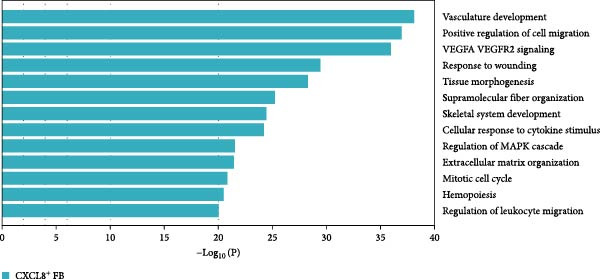
(d)

**Figure:**
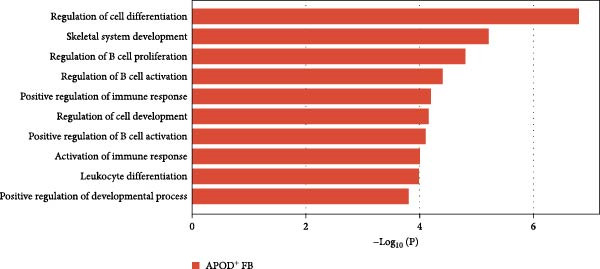
(e)

**Figure:**
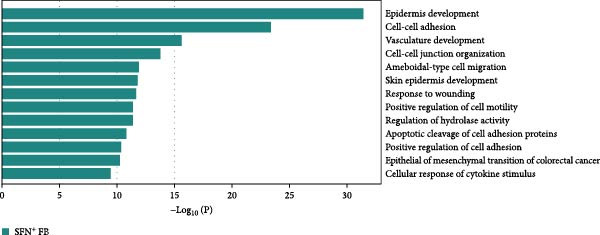
(f)

**Figure:**
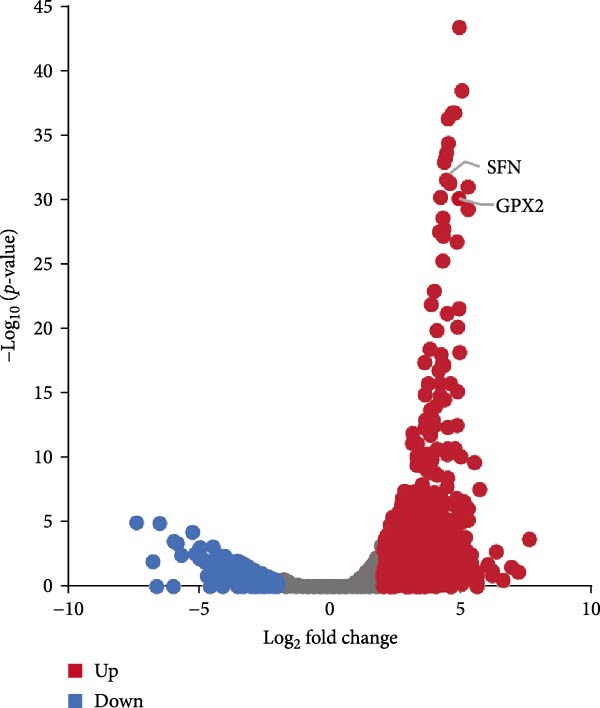
(g)

Five distinct subclusters were identified, exhibiting tissue‐specific distribution patterns yet consistent activation trends from health to disease state (Figure 2b,c and Supporting Information 2: Figure S2a).

APOD^+^ fibroblasts were highly enriched in PB and characterized by features of skeletal system development and cell differentiation (Figure 2e). Further DEG analysis revealed that the expression of osteogenesis‐related genes was upregulated in PB [17–21] (Supporting Information 2: Figure S2b), suggesting a potential role in mediating the imbalance between bone formation and resorption during severe peri‐implantitis. Moreover, it also demonstrated B cell activation in PB, explaining the significant increase of the subcluster under peri‐implantitis.

PG were dominated by CXCL8^+^ fibroblasts, which exhibited enhanced immune response signatures and endothelial interaction potential (Figure 2d), while they were virtually undetectable in alveolar bone. Known for recruiting immune cells [22, 23], CXCL8 has been linked not only to immune activity but also to angiogenesis in some deseases [24–26], suggesting a key role for CXCL8^+^ fibroblast in mediating inflammation and vascular growth specifically in disease‐affected gingival tissue.

Notably, a shared feature of both tissues was the abundance of SFN^+^ fibroblast. Interestingly, this subcluster co‐expressed classical fibroblast markers and epithelial markers (*KRT16*, *KRT4*) (Supporting Information 2: Figure S2c), potentially representing a transitional cell type during epithelial mesenchymal transition (EMT) in peri‐implantitis. Further DEG analysis supported this hypothesis, with elevated expression of EMT markers (*SFN*, *GPX2*) & nbsp [27, 28], and enrichment of cell–cell adhesion and EMT‐related pathways (Figure 2g,f).

Periodontal ligament fibroblast (PLF), identified by extracellular matrix marker *ASPN* [29], were notably reduced in diseased tissues. PLF has been suggested to be a source of osteoblast for the remodeling of alveolar bone [30]. This decline suggests an imbalance in osteogenic function during inflammation, which aligns with the pathogenesis of peri‐implantitis.

Overall, our data reveal a complex landscape of fibroblast diversity between gingival and alveolar bone tissues, highlighting inflammation responses, osteogenesis, and vasculature development.

### 3.3. Endothelial Cells Exhibit a Close Relationship With Both Innate and Adaptive Immunity

Recent studies have confirmed the important roles of endothelial cells in coordinating inflammatory responses [10, 31]. Considering their potential role in shaping the local inflammatory microenvironment, we further investigated the endothelial cell compartment. (Figure 3a–c and Supporting Information 3: Figure S3a,c).

Figure 3Distinct subclusters of the endothelial cell. (a) UMAP of 2790 endothelial cells as in Figure 1a, annotated and colored by the sample type of origin (HG, PG) and subclustering. Endo：Endothelial. (b) Proportion of each cluster in HB, PB, HG, PG. (c) Dot plot depicting the expression of cluster‐defining genes and percentage of cells expressing each gene for the six subclusters of endothelial cells, respectively. Expression values are normalized and scaled averages. (d) Gene enrichment analysis of IL18BP^+^ endothelial associated with genes upregulated between HG versus PG. (e) Gene enrichment analysis of IL6^+^ endothelial associated with genes upregulated between HG versus PG. (f) Gene enrichment analysis of SEMAG3^+^ endothelial associated with genes upregulated between HG versus PG. (g) Violin plot showing the expression of *CXCL12* in each subcluster. Differentially expressed gene criteria included FDR < 0.1 and adjusted *p* value < 0.05. (h) Volcano plot depicting DEGs of IL6^+^ Endothelial between HG and PG. Red points represent genes with FDR < 0.1, log_2_ (fold change) > 2.0 and adjusted *p* value < 0.05 in PG.

**Figure:**
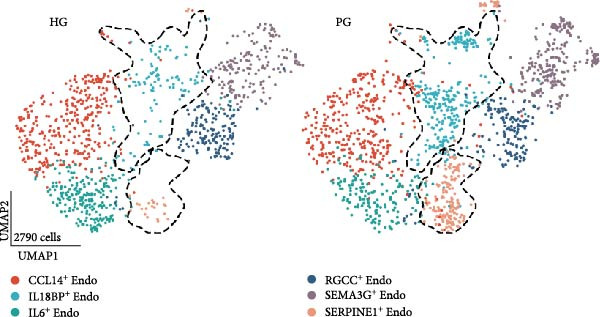
(a)

**Figure:**
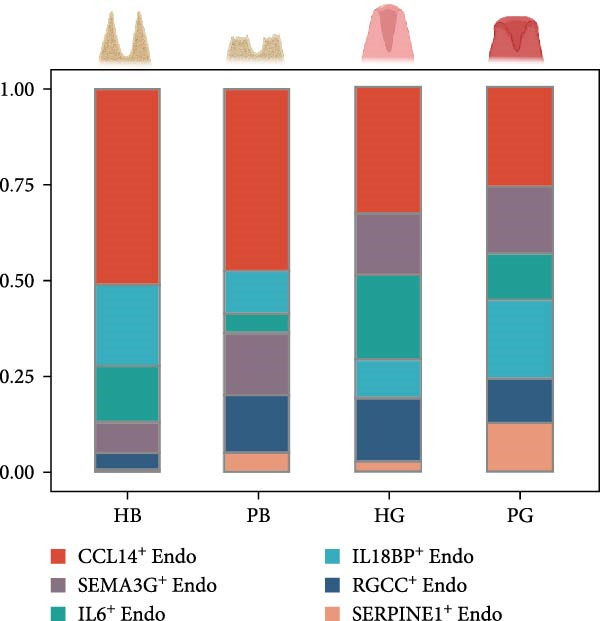
(b)

**Figure:**
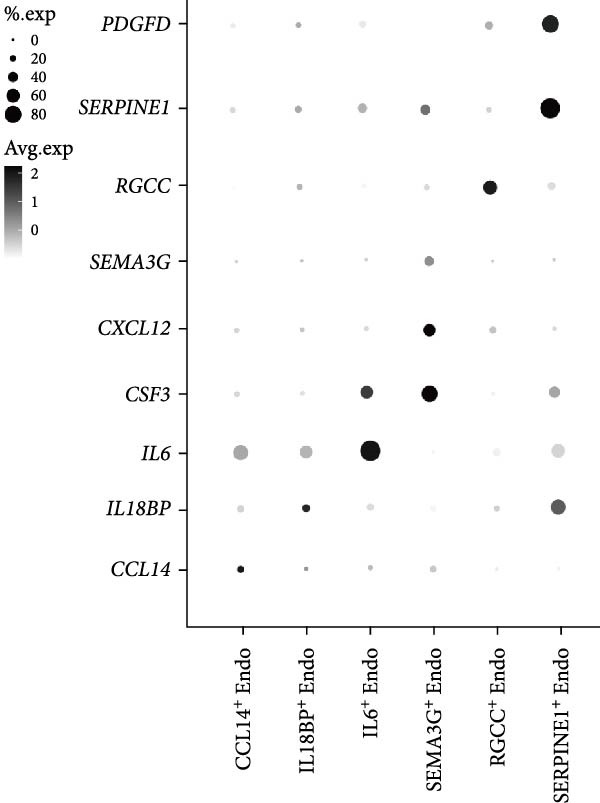
(c)

**Figure:**
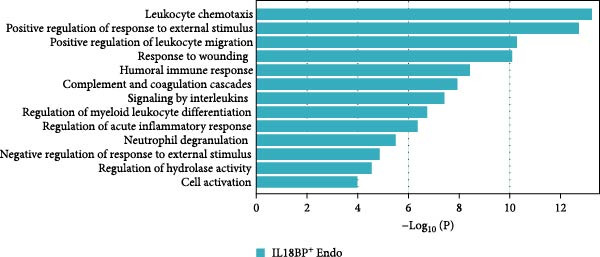
(d)

**Figure:**
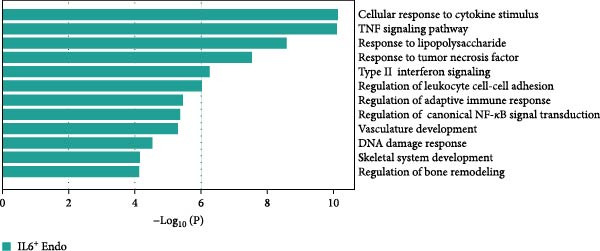
(e)

**Figure:**
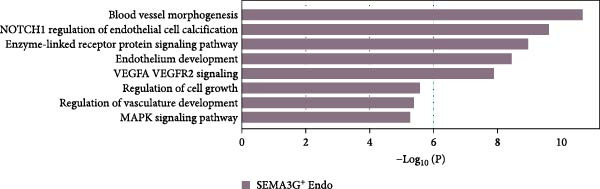
(f)

**Figure:**
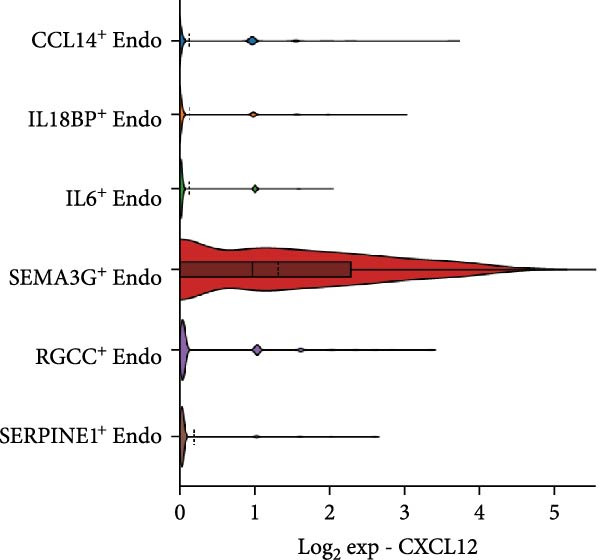
(g)

**Figure:**
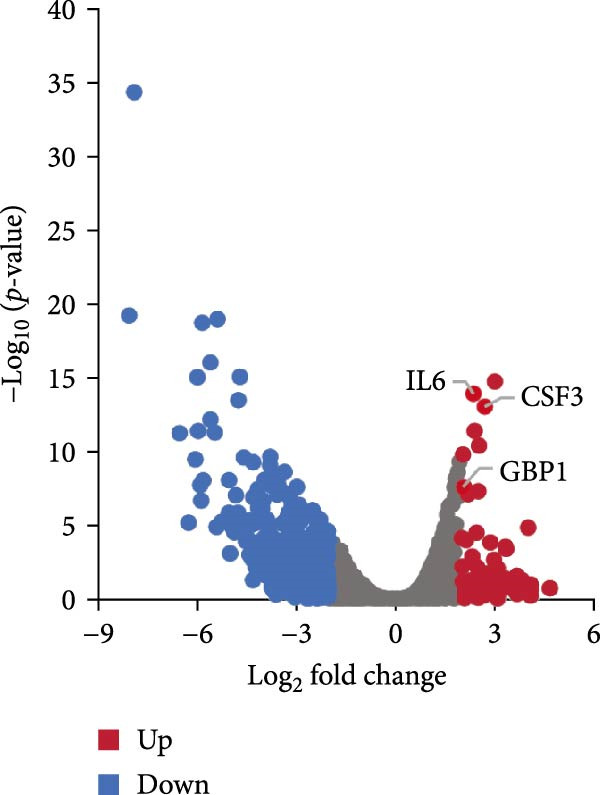
(h)

Among the endothelial subclusters, SERPINE1^+^ endothelial cells were significantly expanded in PG. These cells are characterized by high expression of SERPINE1, a plasminogen activator inhibitor known to regulate inflammation and angiogenesis [32]. Prior studies indicated that endothelial cells in other tissues can express *SERPINE1* in response to endotoxins, contributing to a prothrombotic state by reducing fibrin degradation [33, 34]. Therefore, we speculate that the upregulation of this subcluster may leads to local microthrombosis and necrosis in the gingival, ultimately exacerbating the inflammatory response.

IL18BP^+^ endothelial cells were also increased in the PG. Gene enrichment analysis revealed their role in promoting chemotaxis and the migration of immune cells, especially those involved in the innate immune response (Figure 3d). Several DEGs, including *CXCL8*, *CXCL17*, *CCL24*, *CCL20*, *CCR1*, and *CCR10*, were also increased in the PG, consistent with the immune‐recruiting function. (Supporting Information 3: Figure S3b).

GO analysis revealed that IL6^+^ endothelial cells were also linked to immune functions, with a gene signature of response to lipopolysaccharide and regulation of the adaptive immune response. Although the proportion of IL6^+^ endothelial cell decreased in the inflammatory state, we found that the expression of inflammation‐associated genes were upregulated in the PG (Figure 3h), consisting with GO analysis [35–37] (Figure 3e).

Additionally, SEMAG3^+^ endothelial cells displayed a gene signature consistent with the function of mesenchymal stem cells, including blood vessel morphogenesis and endothelial cell differentiation (Figure 3f). This subcluster also expressed high level of *CXCL12* (Figure 3g), a marker for perivascular mesenchymal stem cells [38]. Given that vascularization and vasodilatation increasing during inflammation [39], the modest increase in SEMAG3^+^ endothelial cells observed in peri‐implantitis suggests their involvement in inflammation‐driven vascular remodeling.

Overall, the gene expression profile of endothelial cells involves not only the matrix organization but also immune processes through direct chemotaxis and the recruitment of immune cells, as well as indirectly through angiogenesis, including endothelial cell migration and proliferation.

### 3.4. The Immune Landscapes of Alveolar Bone and Gingival Tissues From Peri‐Implantitis Canines Showed Heterogeneity

Peri‐implant inflammation is a common complication after implantation, mainly manifested as chronic inflammation of the peri‐implant tissue [40, 41]. To better understand the immune dynamics underlying this condition, we next aimed to characterize immune cells in both tissues at the single‐cell level. While the same immune cell subclusters were identified in both tissues, their proportions varied significantly.

In the gingiva, T cell predominated in the HG but did not increase in the PG, possibly due to a sharp increase in myeloid cells (Supporting Information 4: Figure S4a). In contrast, alveolar bone tissues exhibited an increase trend, as confirmed by flow cytometry. (Figure 4a,d). Myeloid cell populations were most abundant in HB, with expansion in gingival tissues but a decrease in alveolar bone tissue (Supporting Information 4: Figure S4a). However, flow cytometry revealed an increase in both tissues (Figure 4c,f), reflecting a potential limitation of this method in capturing myeloid transcriptomes [7]. Notably, B and plasma cells, particularly the latter, were significantly expanded in peri‐implantitis across both tissues, which was consistent with previous studies and corroborated by the flow cytometry results [42] (Supporting Information 4: Figure S4a and Figure 4b,e).

Figure 4Flow cytometry was used to verify the changes in immune cells. (a–c) Representative flow‐cytometry scatter plots from an independent HG, PG, HB, PB cohort. Cells were gated from single/live and stained with CD45/CD3 (a), CD45/B220 (b), and CD11 b/Ly6G (c). (d–f) Bar graphs demonstrate percentage of expression of CD45^+^CD3^+^(d), CD45^+^B220^+^(e), and CD11b^+^Ly6G^+^(f) (*n* = 3/group, error bars represent SEM,  ^∗^ *p*  < 0.05,  ^∗∗^ *p*  < 0.01,  ^∗∗∗^ *p*  < 0.001,  ^∗∗∗∗^ *p*  < 0.0001 as determined by unpaired two‐tailed student’s *t* test).

**Figure:**
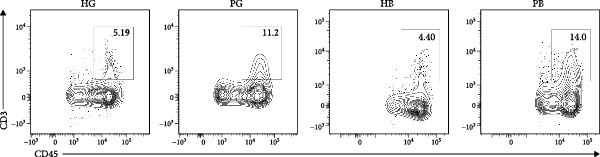
(a)

**Figure:**
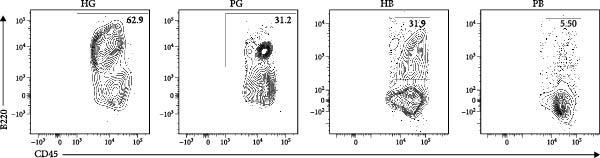
(b)

**Figure:**
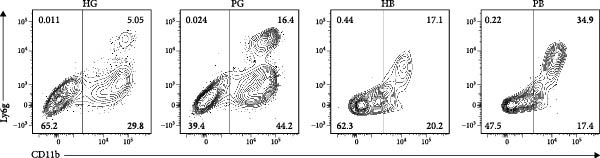
(c)

**Figure:**
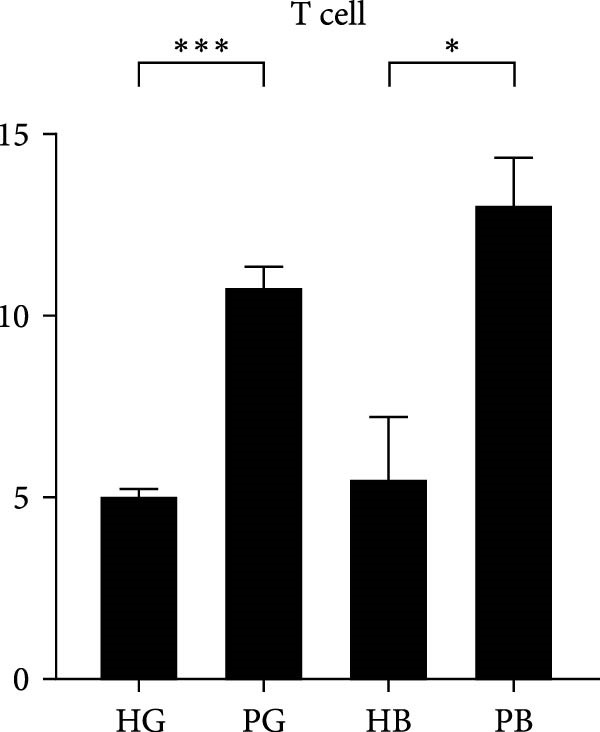
(d)

**Figure:**
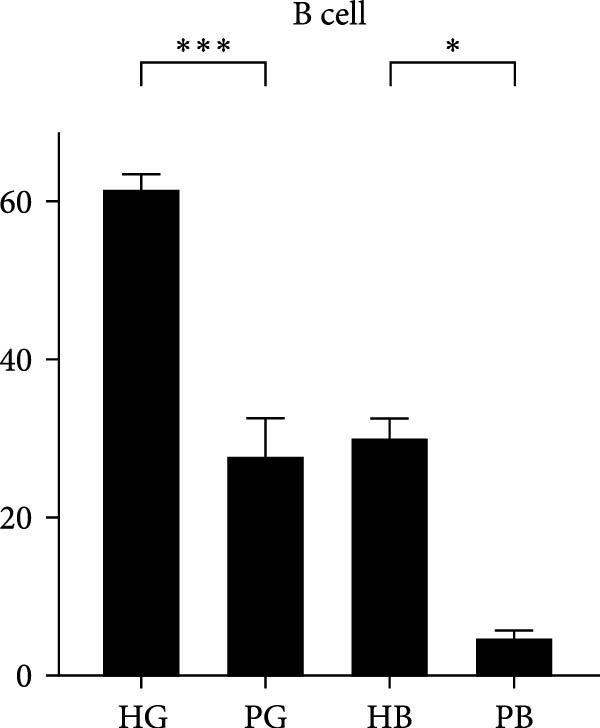
(e)

**Figure:**
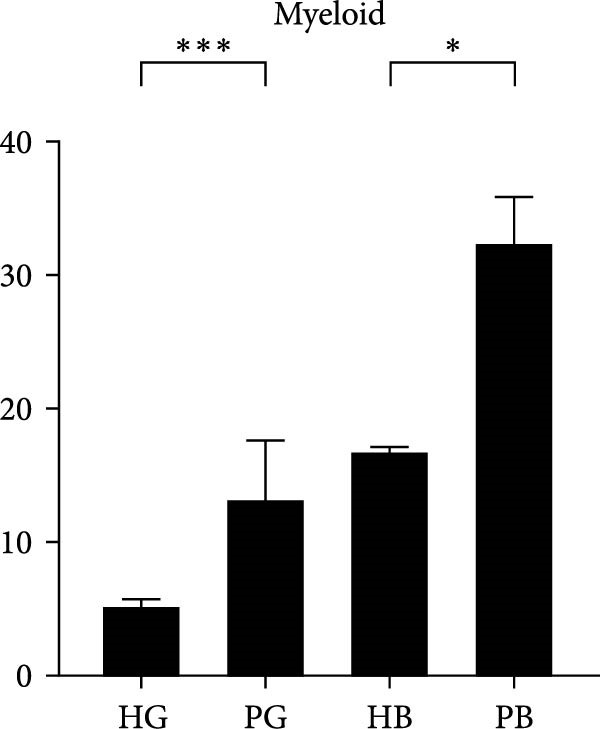
(f)

Given the central role of T and B cells in chronic inflammation [43–45], we next characterized the T cell populations and identified 10 subclusters (Figure 5a,b). T_naive declined in both the PG and PB, indicating a T cell‐activated state. However, T cell subclusters displayed different trends within alveolar bone and gingival tissues. In alveolar bone tissue, almost all the T cell subclusters presented a proportional increase, mainly in T_IL2 and T_FOS, in addition to T_cyto, Th17, ILC2, and NK cells, which indicated a state of T cell activation and tissue destruction. Moreover, although the number of Treg increased, the Th17/Treg ratio still increased in the inflammatory state. In gingival tissue, T_SLPI showed a predominant increase, along with Th17, NK, and ILC2s. In contrast, Tfh cells were decreased (Figure 5c).

Figure 5Distinct subclusters of the immune cell clusters. (a) UMAP of T cells shown in Figure 1a, annotated and colored by the sample type of origin (HB, PB, HG, PG) and subclustering. (b) Dot plots depicting the expression of cluster‐defining genes and the percentage of cells expressing each gene among the subclusters of T cells. The expression values are normalized and scaled average. (c) Proportion of each T cell subcluster in the HB, PB, HG, and PG. (d) UMAP of B cells as in Figure 1a, annotated and colored by the sample type of origin (HB, PB, HG, PG) and subclustering. (e) Violin plots showing the expression of the markers of B cell subclusters. Differentially expressed gene criteria included FDR < 0.1, adjusted *p* value < 0.05 and Log_2_ fold change > 2. (f) Heatmap showing the pathways enriched in all the T cell subclusters in both tissues under peri‐implantitis.

**Figure:**
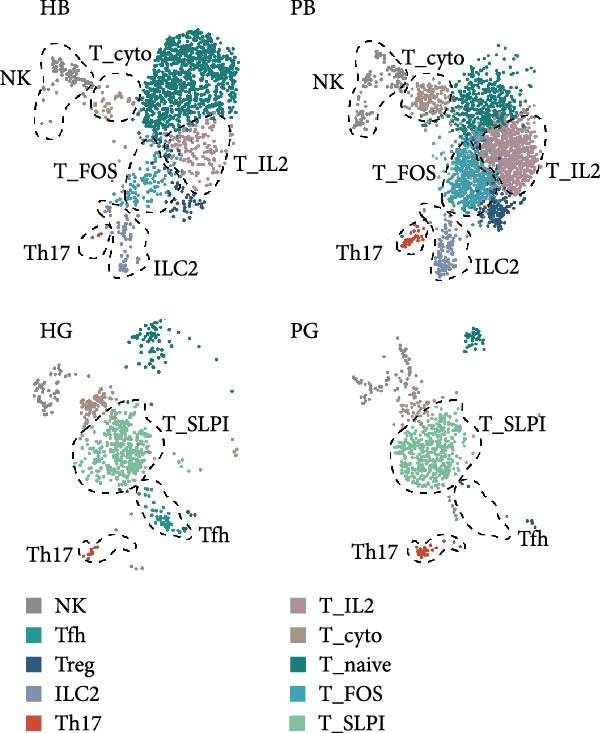
(a)

**Figure:**
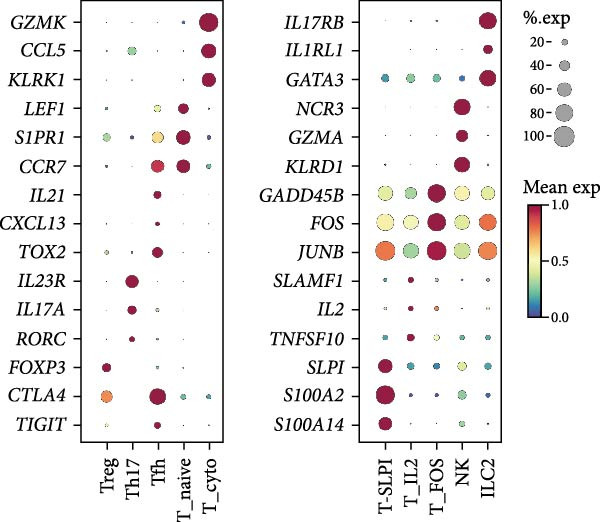
(b)

**Figure:**
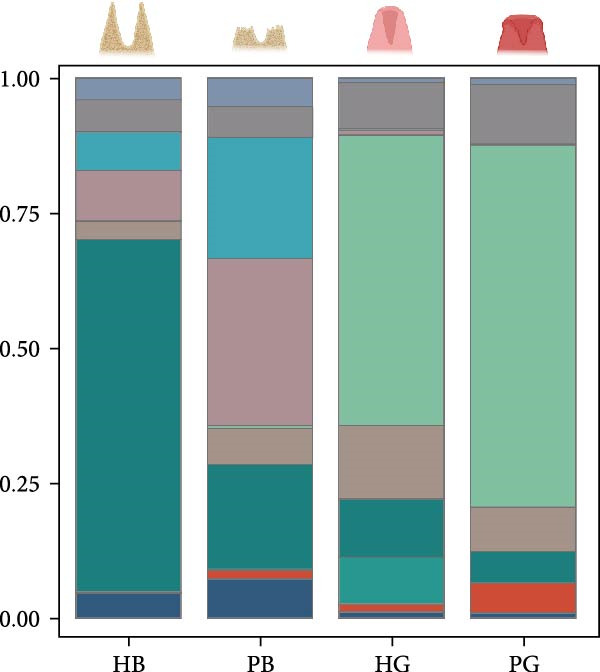
(c)

**Figure:**
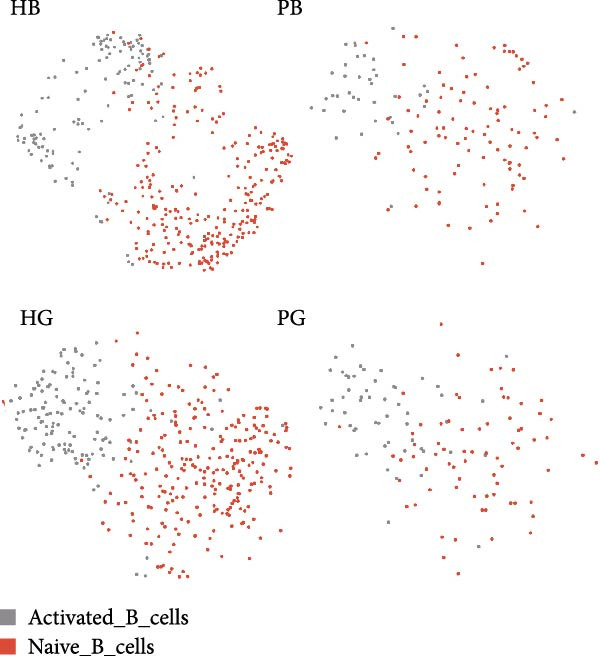
(d)

**Figure:**
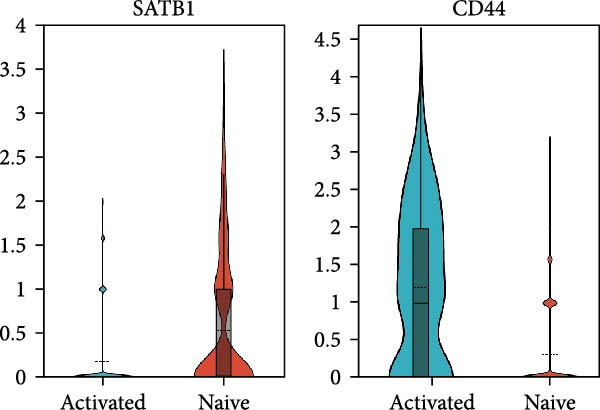
(e)

**Figure:**
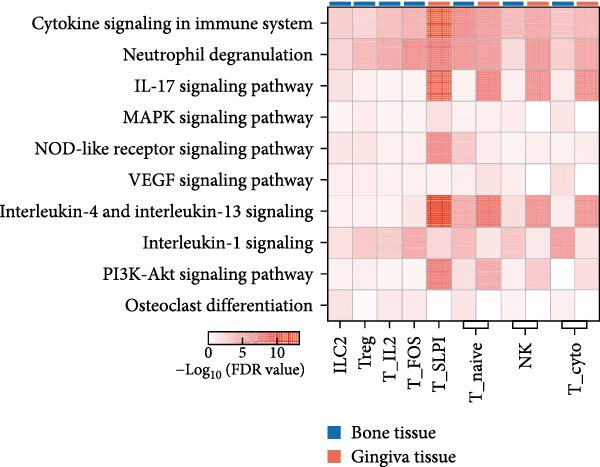
(f)

The alterations in the proportions of T cell subclusters reflected an inflammatory state in both alveolar bone and gingival tissues, and gene enrichment analysis confirmed this phenomenon. Various signaling pathways, including cytokine signaling in the immune system, neutrophil degranulation, the IL‐17 signaling pathway, the MAPK signaling pathway, and the NOD‐like receptor signaling pathway were were upregulated in the inflamed groups across most T cell subclusters (Figure 5f and Supporting Information 4: Figure S4d). These findings underscore the pivotal role of T cells in driving peri‐implantitis through diverse inflammatory pathways.

Finally, we identified two B cell subclusters, activated B cell and naive B cell (Figure 5d,e). A general decline in B cell numbers was observed, likely due to their differentiation into plasma cells, which were markedly increased under peri‐implantitis in both tissues (Figure 1c). The plasma cell‐based state suggests that the activation of B cells in peri‐implantitis is quite intense, which is consistent with previous studies [42]. Further differential expression analysis of B cells revealed an enrichment of the metal ion response pathway in activated B cells in alveolar bone tissue, suggesting that metal ions impact disease via activated B cells (Supporting Information 4: Figure S4c). The extreme condition of the oral environment makes even a stable titanium (Ti) implant undergo corrosion and release metal particles into the surrounding microenvironments, compromising tissue integrity and impairing osseointegration. Previous studies showed that the release of Ti particles commences immediately following dental implant insertion [46], with high levels of Ti particles negatively impacting osteoblastogenesis while promoting osteoclast and inflammatory cell activation [47–49]. Our findings reveal that activated B cells in bone tissues upregulate metal ion‐responsive pathways, potentially linking Ti particle exposure to osteoclast dysfunction through the stimulation of osteoblast precursors by NF‐κB ligand (RANKL), a cytokine essential for osteoclast differentiation.

### 3.5. Cell‒Cell Communication in Peri‐Implantitis Canines and Healthy Controls in the Microenvironments of Alveolar Bone and Gingival Tissues

On the basis of the above results, we conclude that peri‐implantitis involves different complex interactions between immune and nonimmune cells in both tissues. To further investigate these interactions, we employed CellPhoneDB to profile cell communication, with a focus on potential immune–nonimmune interactions.

In both alveolar bone and gingival tissues, the cell‒cell interaction landscape was dominated by endothelial cells and fibroblasts (Figure 6a). On the basis of the subclusters related to inflammation that we observed, we further calculated the interaction strengths of ligand‒receptor (L‒R) pairs in our scRNA‐seq dataset and identified interaction pairs displaying significant cell population specificity between nonimmune cells and immune cells.

Figure 6Cell‒cell communication of the subclusters within the HG, PG, HB, and PB. (a) Overview of the cell‒cell interaction heatmaps of HB, PB, HG, and PG. (b) Dot plot showing the interactions between endothelial cells and immune cells in gingival tissue. (c) Dot plot showing the interactions between fibroblasts and immune cells in gingival tissue.

**Figure:**
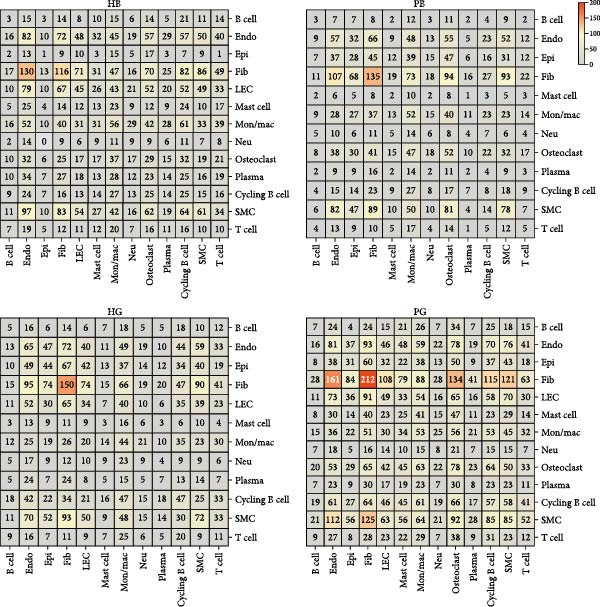
(a)

**Figure:**
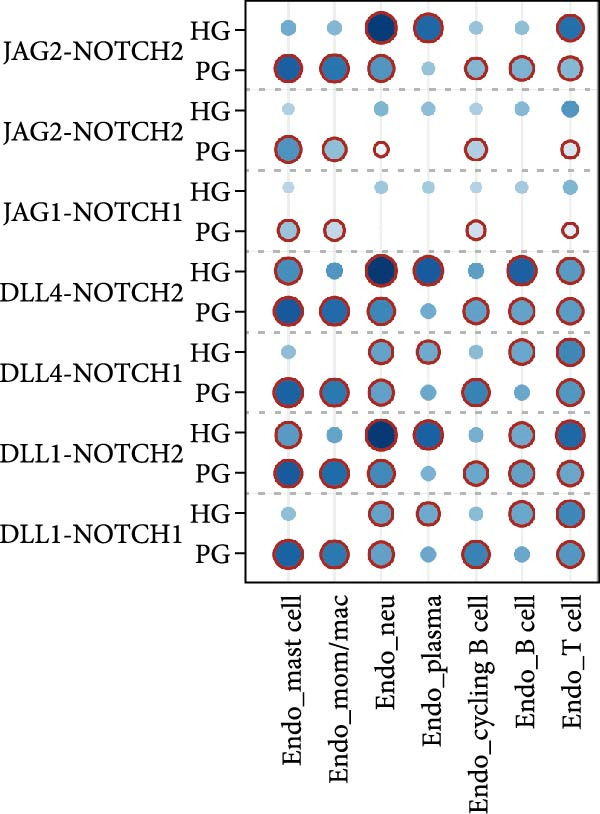
(b)

**Figure:**
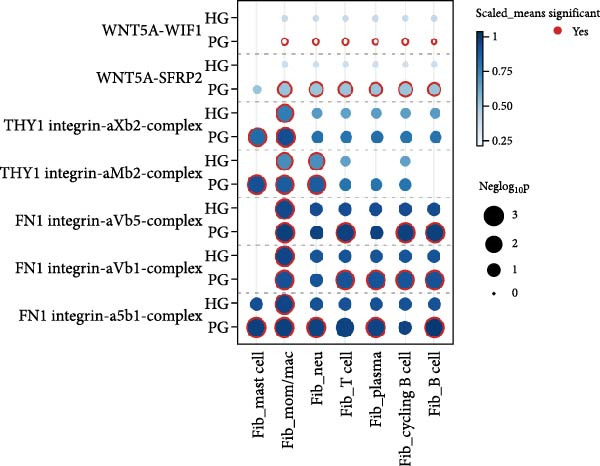
(c)

In the PG group, the endothelial cells presented notably elevated levels of NOTCH ligands, with corresponding receptors expressed by immune cells (Figure 6b and Supporting Information 5: Figure S5b). In addition to the expression of certain chemokines, the WNT signaling pathway is upregulated in fibroblasts during inflammatory states (Figure 6c and Supporting Information 5: Figure S5c). In alveolar bone tissues, we have focused mainly on the interactions between fibroblasts and immune cells. More L–R pairs associated with monocyte and macrophage adhesion and chemotaxis were observed in the inflammatory state, indicating that innate immunity plays a pivotal role in alveolar bone tissue (Supporting Information 5: Figure S5a). This upregulation implies the critical involvement of these pathways in the deterioration of peri‐implantitis, highlighting the importance of understanding their interplay in the pathogenesis of different tissues.

Moreover, we identified a unique fibroblast–myeloid L–R pair: C3–C3AR1. Violin plots revealed that C3 was specifically expressed in APOD^+^ fibroblast, while C3AR1 was predominantly expressed in Mon/Mac (Supporting Information 5: Figure S5d), indicating that the fibroblast–Mon/Mac interaction through C3–C3AR1 signaling is highly related to pathology. Previous studies showed that the cleavage product C3a of complement component C3 supports the recruitment of macrophages [50, 51]. Therefore, we hypothesize that the APOD^+^ fibroblast subcluster may recruit macrophages through the C3–C3AR1 L–R pair under inflammatory conditions, thereby modulating the inflammatory process.

## 4. Discussion

In our study, we provided an unbiased transcriptome‐wide perspective profiling of a total of 35039 single cells from canine gingival and alveolar bone tissues via scRNA‐seq, focusing on the changes of cell types, gene expression, and ligand‐receptor pairs in different tissues during the inflammatory process. These findings advance our understanding of the inflammatory process of peri‐implantitis, enabling the development of specific targeted therapeutic strategies against abnormally expressed key genes, functionally aberrant cell subsets and L–R pairs.

Here, we initially obtained samples from beagle dogs, aiming to acquire healthy controls from authentic peri‐implant tissue instead of nonimplant sites. The differences between the implants used in small animal models and those used in clinical make large animals a better option for studying peri‐implant diseases [52, 53]. Compared with other species, the canine model is a preferred large‐animal model for studying peri‐implant disease, given its higher incidence of natural periodontal disease and similarities to human periodontitis lesions [54].

Our findings demonstrated that among the fibroblast populations, CXCL8^+^ fibroblasts, SFN^+^ fibroblasts and APOD^+^ fibroblasts were more common in peri‐implantitis canines than in healthy controls. These fibroblasts exhibited immune‐related functions. CXCL8 is a strong chemokine for neutrophil, lymphocyte, and monocyte recruitment [22, 23], and a high concentration of CXCL8 was reported to be associated with strong angiogenesis [24]. Our study revealed that angiogenesis and the response to chronic inflammation may be mediated by CXCL8^+^ fibroblasts, which are specific to gingival tissue in peri‐implantitis. Additionally, SFN^+^ Fib subcluster was found to be highly expressed in inflammatory state and to be actively involved in cell‒cell adhesion and EMT in both gingival and alveolar bone tissues, as indicated by GO analysis. Type 2 EMT is linked with tissue repair responses such as fibrosis, and the involvement of numerous molecules and cells in EMT during wound healing highlights the complexity of tissue repair mechanisms [55]. These findings raise new possibilities for the disease progression of peri‐implantitis.

For the endothelial population, we identified two subclusters highly associated with inflammation, including IL18BP^+^ endothelial cells and IL6^+^ endothelial cells. IL18BP^+^ endothelial cells exhibited stronger chemotactic properties, likely enhancing immune cell migration, particularly in humoral immunity. In contrast, IL6^+^ endothelial cells presented gene patterns related to the lipopolysaccharide response and adaptive immunity regulation. Researches have indicated that endothelial cells have various immune functions, such as cytokine secretion and immune modulation, indicating that they are integral immune players [56–58]. Our findings support the view that IL18BP^+^ endothelial and IL6^+^ endothelial cells may significantly contribute to immune system function.

Immune cells were observed with a particular focus on T cells and B cells. The increase of the proportion of these lymphocytes showed a character of adaptive immune response in peri‐implantitis. Interestingly, GO analysis revealed that the response to metal ions was particularly enriched in activated B cells in alveolar bone tissue, which suggests that the influence on peri‐implantitis via metal ions was induced by activated B cells. Multiple studies have focused on the influence of metal on the deterioration of peri‐implantitis [47, 59, 60], and our study further support the hypothesis that metal ions may have an impact on the immune system and ultimately lead to alveolar bone loss.

Cell–cell communication further revealed strong interactions between nonimmune and immune cells. Tissue‐specific heterogeneity was evident, with endothelial and immune cells active in the NOTCH pathway in the gingival, whereas fibroblasts dominated the WNT pathway, which is typical of inflammation [61–63]. In alveolar bone, innate immune cells such as monocytes and macrophages have a significant impact. This highlights the importance of cell communication in understanding peri‐implant inflammation and points to potential targeted therapies for peri‐implantitis. In addition, we also found a unique L‒R pair in alveolar bone tissue: C3 (APOD^+^ fibroblasts)–C3AR1 (monocytes/macrophages). These results further substantiate our hypothesis that there could be an increased interaction between inflammation‐promoting fibroblasts and the monocytes/macrophages subtype in peri‐implantitis.

Despite the notable advantages of using canines as the animal model in this study, it is important to acknowledge the inherent limitations relative to human peri‐implantitis. Specifically, the experimental model of peri‐implantitis in our study was only induced by mechanical stimulation, in the absence of the causative pathogens present in naturally occurring human peri‐implantitis.

In our study, we discovered an increase in inflammation‐associated subclusters in both gingival and alveolar bone tissues affected by peri‐implantitis, indicating an intense inflammatory reaction. This study highlighted the roles of IL18BP^+^ endothelial, IL6^+^ endothelial, CXCL8^+^ fibroblast, APOD^+^ fibroblast and SFN^+^ fibroblasts, as well as their interactions with immune cells. These distinct cell types and communications in gingival and alveolar bone tissues in peri‐implantitis were illuminated, providing novel insights into tissue‐specific differences. Further research is needed to clarify the specific functions of these cells and the key pathways involved in disease progression and treatment in human peri‐implantitis tissues.

## 5. Conclusions

Our study delineates tissue‐specific inflammatory landscapes of canine peri‐implantitis at single‐cell resolution. The expansion of IL6^+^/IL18BP^+^ endothelial cells and CXCL8^+^ fibroblasts in gingiva, alongside APOD^+^ fibroblast‐driven C3–C3AR1 signaling in alveolar bone, highlights distinct microenvironmental reprogramming between soft and hard tissues. These findings not only identify potential therapeutic targets but also validate the translational relevance of the canine model for peri‐implantitis research.

## Disclosure

All authors gave final approval and agreed to be accountable for the submitted version.

## Conflicts of Interest

The authors declare no conflicts of interest.

## Author Contributions

All the authors have made substantial contributions to the conception and design of the study. Ming Wang and Daner Wu conceived the idea; they also critically revised the manuscript with Chunhui Liao. Dong Zhang conducted additional revisions to the entire manuscript and prepared the response letter during the re‐submission process. Yixin Xia and Ningbo Geng constructed the canine peri‐implantitis models and collected the samples. Yu Chen prepared the samples and performed the scRNA‐seq. Ming Wang, Daner Wu, and Chunhui Liao analyzed the data. Songling Chen and Wei Peng carried out the statistical analysis and critically revised the manuscript. Ming Wang, Dong Zhang, Chunhui Liao, and Daner Wu contributed equally to this work.

## Funding

This research was financed by the Natural Science Foundation of Guangdong Province,China (Nos. 2021A1515010806 and 2022A1515010809) and the Guangzhou Municipal Health Technology Project, China (No. 205151017014).

## Supporting Information

Additional supporting information can be found online in the Supporting Information section.

## Supporting information


**Supporting Information 1** Figure S1 related to Figure 1. (a) Violin plots showing the number of features, RNA counts and percentage of mitochondrial transcripts found in HB, PB, HG, PG samples prior to quality control. (b) UMAPs are the same as those in Figure 1b but are colored according to the expression of key cell‐type markers. Log_2_ Exp: Log_2_ expression. (c) Absolute proportions of the clusters of HB, PB, HG, and PG. (c) The proportion changes in each cluster from the healthy state to the inflammatory state. (d) Left: Proportion of each cluster of HB, PB. Right: Proportion of each cluster of HG, PG. (e) Left: The UMAPs as in Figure 1b but colored by immune cells and nonimmune cells. Right: The proportion of immune cells and nonimmune cells in HG, PG, HB, and PB.

**Supporting Information 2** Figure S2 related to Figure 2. (a) Absolute proportions of the clusters of HB, PB, HG, and PG in the fibroblast population. (b) Volcano plot depicting the increased expression of osteogenesis‐related genes in the PB of APOD^+^Fib. Red points represent genes with FDR < 0.1, log_2_ (fold change) > 2.0 and adjusted *p* value < 0.05 in PB. Blue points represent genes with FDR < 0.1, log_2_ (fold change) < −2.0 and adjusted *p* value < 0.05 in PB. (c) Violin plot showing the genes highly expressed in SFN^+^Fib.

**Supporting Information 3** Figure S3 related to Figure 3. (a) UMAP of endothelial cells as in Figure 1a, annotated and colored by the sample type of origin (HB, PB) and subclustering. (b) Volcano plot depicting the increased expression of cytokine genes in the PG of IL18BP^+^ endothelial cells. Red points represent genes with log_2_ (fold change) > 2.0 and adjusted *p* value <0.05 in the PG. (c) Absolute proportion of the clusters of HB, PB, HG, and PG in the endothelial population.

**Supporting Information 4** Figure S4 related to Figure 5. (a) Proportion of T cells, B cells/plasma and myeloid cells in the HB, PB, HG, and PG. (b) Upper panel: Proportion of the subclusters of B cells. Lower panel: Absolute proportion of the subclusters of B cells. (c) Enrichment gene analysis of activated B cells in HB versus PB. (d) Heatmap showing the pathways downregulated in all the T cell subclusters in both tissues.

**Supporting Information 5** Figure S5 related to Figure 6. (a) Dot plot showing the interactions between fibroblasts and immune cells in bone tissue. (b) Dot plot showing the interactions between endothelial cells and immune cells in gingival tissue. (c) Dot plots showing the interactions between fibroblasts and immune cells in gingival tissue. (d) Violin plots showing the expression of C3 and C3AR1 in fibroblasts and immune cells.

## Data Availability

The raw sequencing data that support the findings of this study are available in the GEO datasets (GSE281266). The authors declare that all other data supporting the findings of this study are available within the article and its supporting information files or are available from the corresponding author upon request.

## References

[1] Smeets R. , Henningsen A. , and Jung O. , et al.Definition, Etiology, Prevention and Treatment of Peri-Implantitis—A Review, Head & Face Medicine. (2014) 10, no. 1, 10.1186/1746-160X-10-34, 2-s2.0-84907406817, 34. 25185675 PMC4164121

[2] Salvi G. E. , Cosgarea R. , and Sculean A. , Prevalence and Mechanisms of Peri-Implant Diseases, Journal of Dental Research. (2017) 96, no. 1, 31–37, 10.1177/0022034516667484, 2-s2.0-85007202544. 27680028

[3] Schwarz F. , Jepsen S. , Obreja K. , Galarraga-Vinueza M. E. , and Ramanauskaite A. , Surgical Therapy of Peri-Implantitis, Periodontology 2000. (2022) 88, no. 1, 145–181, 10.1111/prd.12417. 35103328

[4] Figuero E. , Graziani F. , Sanz I. , Herrera D. , and Sanz M. , Management of Peri-Implant Mucositis and Peri-Implantitis, Periodontology 2000. (2014) 66, no. 1, 255–273, 10.1111/prd.12049, 2-s2.0-84906057257. 25123773

[5] Heitz-Mayfield L. J. , Aaboe M. , and Araujo M. , et al.Group 4 ITI Consensus Report: Risks and Biologic Complications Associated With Implant Dentistry, Clinical Oral Implants Research. (2018) 29, no. S16, 351–358, 10.1111/clr.13307, 2-s2.0-85055023401. 30328181

[6] Li J. , Ye L. J. , and Dai Y. W. , et al.Single-Cell Analysis Reveals a Unique Microenvironment in Peri-Implantitis, Journal of Clinical Periodontology. (2024) 51, no. 12, 1665–1676, 10.1111/jcpe.13982. 38566468 PMC11651718

[7] Williams D. W. , Greenwell-Wild T. , and Brenchley L. , et al.Human Oral Mucosa Cell Atlas Reveals a Stromal-Neutrophil Axis Regulating Tissue Immunity, Cell. (2021) 184, no. 15, 4090–4104, 10.1016/j.cell.2021.05.013. 34129837 PMC8359928

[8] Giro G. , Tebar A. , Franco L. , Racy D. , Bastos M. F. , and Shibli J. A. , Treg and TH17 Link to Immune Response in Individuals With Peri-Implantitis: A Preliminary Report, Clinical Oral Investigations. (2021) 25, no. 3, 1291–1297, 10.1007/s00784-020-03435-w. 32594309

[9] Mo J. , Lai Y. , and Huang Q. , et al.Single-Cell Sequencing Identifies Inflammation-Promoting Fibroblast-Neutrophil Interaction in Peri-Implantitis, Journal of Clinical Periodontology. (2024) 51, no. 2, 196––208, 10.1111/jcpe.13912. 38088448

[10] Niklander S. , Bordagaray M. J. , Fernández A. , and Hernández M. , Vascular Endothelial Growth Factor: A Translational View in Oral Non-Communicable Diseases, Biomolecules. (2021) 11, no. 1, 10.3390/biom11010085, 85. 33445558 PMC7826734

[11] du Sert N. P. , Ahluwalia A. , and Alam S. , et al.Reporting Animal Research: Explanation and Elaboration for the ARRIVE Guidelines 2.0, PLOS Biology. (2020) 18, no. 7, 10.1371/journal.pbio.3000411. PMC7360025 32663221

[12] Xia Y. , Geng N. , and Ren J. , et al.Regulation of Endothelial Cells on the Osteogenic Ability of Bone Marrow Mesenchymal Stem Cells in Peri-Implantitis, Tissue and Cell. (2023) 81, 10.1016/j.tice.2023.102042, 102042. 36812664

[13] Dudziak D. , Heger L. , and Agace W. W. , et al.Guidelines for Preparation and Flow Cytometry Analysis of Human Nonlymphoid Tissue DC, European Journal of Immunology. (2025) 55, no. 1, 10.1002/eji.202250325. PMC11739683 39668411

[14] Zhu G. Q. , Tang Z. , and Huang R. , et al.CD36^+^ Cancer-Associated Fibroblasts Provide Immunosuppressive Microenvironment for Hepatocellular Carcinoma via Secretion of Macrophage Migration Inhibitory Factor, Cell Discovery. (2023) 9. 10.1038/s41421-023-00529-z PMC9988869 36878933

[15] Tirosh I. , Izar B. , and Prakadan S. M. , et al.Dissecting the Multicellular Ecosystem of Metastatic Melanoma by Single-Cell RNA-Seq, Science. (2016) 352, no. 6282, 189–196, 10.1126/science.aad0501, 2-s2.0-84963614956. 27124452 PMC4944528

[16] Liu L. , Chen Y. , and Wang L. , et al.Dissecting B/Plasma Cells in Periodontitis at Single-Cell/Bulk Resolution, Journal of Dental Research. (2022) 101, no. 11, 1388–1397, 10.1177/00220345221099442. 35620808

[17] Muruganandan S. , Govindarajan R. , McMullen N. M. , and Sinal C. J. , Chemokine-Like Receptor 1 Is a Novel Wnt Target Gene That Regulates Mesenchymal Stem Cell Differentiation, Stem Cells. (2017) 35, no. 3, 711–724, 10.1002/stem.2520, 2-s2.0-85001638728. 27733019

[18] Yukata K. , Shukunami C. , and Matsui Y. , et al.Chondromodulin is Necessary for Cartilage Callus Distraction in Mice, PLoS ONE. (2023) 18, no. 2, 10.1371/journal.pone.0280634. PMC9934371 36795722

[19] Hsieh J.-Y. , Fu Y.-S. , Chang S.-J. , Tsuang Y.-H. , and Wang H.-W. , Functional Module Analysis Reveals Differential Osteogenic and Stemness Potentials in Human Mesenchymal Stem Cells From Bone Marrow and Wharton’s Jelly of Umbilical Cord, Stem Cells and Development. (2010) 19, no. 12, 1895–1910, 10.1089/scd.2009.0485, 2-s2.0-78149396432. 20367285

[20] Park S.-J. , Bae H.-S. , and Park J.-C. , Osteogenic Differentiation and Gene Expression Profile of Human Dental Follicle Cells Induced by Human Dental Pulp Cells, Journal of Molecular Histology. (2015) 46, no. 1, 93–106, 10.1007/s10735-014-9604-1, 2-s2.0-84925488311. 25520056

[21] Knight M. N. , Karuppaiah K. , and Lowe M. , et al.R-Spondin-2 Is a Wnt Agonist That Regulates Osteoblast Activity and Bone Mass, Bone Research. (2018) 6, no. 1, 10.1038/s41413-018-0026-7, 2-s2.0-85051497037, 24. 30131881 PMC6089978

[22] Cambier S. , Gouwy M. , and Proost P. , The Chemokines CXCL8 and CXCL12: Molecular and Functional Properties, Role in Disease and Efforts Towards Pharmacological Intervention, Cellular & Molecular Immunology. (2023) 20, no. 3, 217–251, 10.1038/s41423-023-00974-6. 36725964 PMC9890491

[23] Palm E. , Demirel I. , Bengtsson T. , and Khalaf H. , The Role of Toll-Like and Protease-Activated Receptors in the Expression of Cytokines by Gingival Fibroblasts Stimulated With the Periodontal Pathogen *Porphyromonas gingivalis* , Cytokine. (2015) 76, no. 2, 424–432, 10.1016/j.cyto.2015.08.263, 2-s2.0-84943819518. 26318255

[24] Rosenkilde M. M. and Schwartz T. W. , The Chemokine System—A Major Regulator of Angiogenesis in Health and Disease, APMIS. (2004) 112, no. 7-8, 481–495, 10.1111/j.1600-0463.2004.apm11207-0808.x, 2-s2.0-10344262597. 15563311

[25] Schminke B. , Vom Orde F. , Gruber R. , Schliephake H. , Bürgers R. , and Miosge N. , The Pathology of Bone Tissue During Peri-Implantitis, Journal of Dental Research. (2015) 94, no. 2, 354–361, 10.1177/0022034514559128, 2-s2.0-84921329844. 25406169 PMC4438728

[26] Ha H. , Debnath B. , and Neamati N. , Role of the CXCL8-CXCR1/2 Axis in Cancer and Inflammatory Diseases, Theranostics. (2017) 7, no. 6, 1543–1588, 10.7150/thno.15625, 2-s2.0-85018481620. 28529637 PMC5436513

[27] Ye S.-P. , Yu H.-X. , and Lu W.-J. , et al.Stratifin Promotes Hepatocellular Carcinoma Progression by Modulating the Wnt/*β*-Catenin Pathway, International Journal of Genomics. (2023) 2023, no. 1, 10.1155/2023/9731675, 9731675. 37587914 PMC10427227

[28] Peng F. , Xu Q. , and Jing X. , et al.Promotes EMT and Metastasis in Non-Small Cell Lung Cancer by Activating PI3K/AKT/mTOR/Snail Signaling Axis, FASEB BioAdvances. (2023) 5, no. 6, 233–250, 10.1096/fba.2022-00045. 37287867 PMC10242197

[29] Lu E. M. , Hobbs C. , Dyer C. , Ghuman M. , and Hughes F. J. , Differential Regulation of Epithelial Growth by Gingival and Periodontal Fibroblasts *In Vitro* , Journal of Periodontal Research. (2020) 55, no. 6, 859–867, 10.1111/jre.12778. 32885443

[30] Nomura Y. , Ishikawa M. , and Yashiro Y. , et al.Human Periodontal Ligament Fibroblasts are the Optimal Cell Source for Induced Pluripotent Stem Cells, Histochemistry and Cell Biology. (2012) 137, no. 6, 719–732, 10.1007/s00418-012-0923-6, 2-s2.0-84865114978. 22327794

[31] Majolée J. , Kovačević I. , and Hordijk P. L. , Ubiquitin-Based Modifications in Endothelial Cell-Cell Contact and Inflammation, Journal of Cell Science. (2019) 132, no. 17, 10.1242/jcs.227728, 2-s2.0-85071777253. 31488505

[32] Li L. , Li F. , and Xu Z. , et al.Identification and Validation of SERPINE1 as a Prognostic and Immunological Biomarker in Pan-Cancer and in ccRCC, Frontiers in Pharmacology. (2023) 14, 10.3389/fphar.2023.1213891, 1213891. 37680718 PMC10482042

[33] Paulus P. , Jennewein C. , and Zacharowski K. , Biomarkers of Endothelial Dysfunction: Can They Help us Deciphering Systemic Inflammation and Sepsis?, Biomarkers. (2011) 16, no. 1, S11–S21, 10.3109/1354750X.2011.587893, 2-s2.0-79959854675. 21707440

[34] Morrow G. B. and Mutch N. J. , Present, and Future Perspectives of Plasminogen Activator Inhibitor 1 (PAI-1), Seminars in Thrombosis and Hemostasis. (2023) 49, no. 3, 305–313, 10.1055/s-0042-1758791. 36522166

[35] Garcia-Bonilla L. , Racchumi G. , Murphy M. , Anrather J. , and Iadecola C. , Endothelial CD36 Contributes to Postischemic Brain Injury by Promoting Neutrophil Activation via CSF3, The Journal of Neuroscience. (2015) 35, no. 44, 14783–14793, 10.1523/JNEUROSCI.2980-15.2015, 2-s2.0-84946430617. 26538649 PMC4635129

[36] Kirkby M. , Enosi Tuipulotu D. , Feng S. , Lo Pilato J. , and Man S. M. , Guanylate-Binding Proteins: Mechanisms of Pattern Recognition and Antimicrobial Functions, Trends in Biochemical Sciences. (2023) 48, no. 10, 883–893, 10.1016/j.tibs.2023.07.002. 37567806

[37] Lumbikananda S. , Srithanyarat S. S. , Mattheos N. , and Osathanon T. , Oral Fluid Biomarkers for Peri-Implantitis: A Scoping Review, International Dental Journal. (2024) 74, no. 3, 387–402, 10.1016/j.identj.2023.11.005. 38065782 PMC11123564

[38] Hsu Y.-M. S. , Greenbaum A. , and Schuettpelz L. G. , et al.CXCL12 Production by Early Mesenchymal Progenitors is Required for Hematopoietic Stem Cell Maintenance, Blood. (2012) 120, no. 21, 510–510, 10.1182/blood.V120.21.510.510.

[39] Sakallıoğlu E. E. , Ayas B. , Sakallıoğlu U. , Açıkgöz G. , and Çağlayan F. , Osmotic Pressure and Vasculature of Gingiva in Periodontal Disease: An Experimental Study in Rats, Archives of Oral Biology. (2006) 51, no. 6, 505–511, 10.1016/j.archoralbio.2005.11.004, 2-s2.0-33747794368. 16376292

[40] Belibasakis G. N. and Manoil D. , Microbial Community-Driven Etiopathogenesis of Peri-Implantitis, Journal of Dental Research. (2021) 100, no. 1, 21–28, 10.1177/0022034520949851. 32783779 PMC7754824

[41] Albrektsson T. , Jemt T. , Mölne J. , Tengvall P. , and Wennerberg A. , On Inflammation-Immunological Balance Theory—A Critical Apprehension of Disease Concepts around Implants: Mucositis and Marginal Bone Loss May Represent Normal Conditions and Not Necessarily a State of Disease, Clinical Implant Dentistry and Related Research. (2019) 21, no. 1, 183–189, 10.1111/cid.12711, 2-s2.0-85059275265. 30592373

[42] Kulakov A. A. , Kogan E. A. , Brailovskaya T. V. , Vedyaeva A. P. , and Zharkov N. V. , Morphological and Molecular-Biological Features of Inflammatory and Regeneratory Processes in Peridont Tissues With Periimplantitis and Periodontitis, Doklady Biochemistry and Biophysics. (2020) 492, no. 1, 142–146, 10.1134/S1607672920030060. 32632592

[43] Li Y. , Ling J. , and Jiang Q. , Inflammasomes in Alveolar Bone Loss, Frontiers in Immunology. (2021) 12, 10.3389/fimmu.2021.691013, 691013. 34177950 PMC8221428

[44] Konieczny J. and Arranz L. , Updates on Old and Weary Haematopoiesis, International Journal of Molecular Sciences. (2018) 19, no. 9, 10.3390/ijms19092567, 2-s2.0-85052646822, 2567. 30158459 PMC6163425

[45] Gruber R. , Osteoimmunology: Inflammatory Osteolysis and Regeneration of the Alveolar Bone, Journal of Clinical Periodontology. (2019) 46, 52–69, 10.1111/jcpe.13056, 2-s2.0-85067562492. 30623453

[46] Silva G. A. F. , Faot F. , Possebon A. P. R. , Da Silva W. J. , and Del Bel Cury A. A. , Effect of Macrogeometry and Bone Type on Insertion Torque, Primary Stability, Surface Topography Damage and Titanium Release of Dental Implants During Surgical Insertion Into Artificial Bone, Journal of the Mechanical Behavior of Biomedical Materials. (2021) 119, 10.1016/j.jmbbm.2021.104515, 104515. 33932754

[47] Asa’ad F. , Thomsen P. , and Kunrath M. F. , The Role of Titanium Particles and Ions in the Pathogenesis of Peri-Implantitis, Journal of Bone Metabolism. (2022) 29, no. 3, 145–154, 10.11005/jbm.2022.29.3.145. 36153850 PMC9511127

[48] Pettersson M. , Pettersson J. , Molin Thorén M. , and Johansson A. , Release of Titanium After Insertion of Dental Implants With Different Surface Characteristics—An Ex Vivo Animal Study, Acta Biomaterialia Odontologica Scandinavica. (2017) 3, no. 1, 63–73, 10.1080/23337931.2017.1399270. 29242814 PMC5724801

[49] Wachi T. , Shuto T. , Shinohara Y. , Matono Y. , and Makihira S. , Release of Titanium Ions From an Implant Surface and Their Effect on Cytokine Production Related to Alveolar Bone Resorption, Toxicology. (2015) 327, 1–9, 10.1016/j.tox.2014.10.016, 2-s2.0-84910014981. 25446332

[50] Davidson S. , Efremova M. , and Riedel A. , et al.Single-Cell RNA Sequencing Reveals a Dynamic Stromal Niche That Supports Tumor Growth, Cell Reports. (2020) 31, no. 7, 10.1016/j.celrep.2020.107628, 107628. 32433953 PMC7242909

[51] Dick J. , Gan P.-Y. , Kitching A. R. , and Holdsworth S. R. , The C3aR Promotes Macrophage Infiltration and Regulates ANCA Production but Does Not Affect Glomerular Injury in Experimental Anti-Myeloperoxidase Glomerulonephritis, PLoS ONE. (2018) 13, no. 1, 10.1371/journal.pone.0190655, 2-s2.0-85040338972. PMC5760037 29315316

[52] Hiyari S. , Wong R. , and Yaghsezian A. , et al.Ligature-Induced Peri-Implantitis and Periodontitis in Mice, Journal of Clinical Periodontology. (2018) 45, no. 1, 89–99, 10.1111/jcpe.12817, 2-s2.0-85034263600. 28921659 PMC5774657

[53] Sun J. , Eberhard J. , and Glage S. , et al.Development of a Peri-Implantitis Model in the Rat, Clinical Oral Implants Research. (2020) 31, no. 3, 203–214, 10.1111/clr.13556. 31701561

[54] Schwarz F. , Sculean A. , Engebretson S. P. , Becker J. , and Sager M. , Animal Models for Peri-Implant Mucositis and Peri-Implantitis, Periodontology 2000. (2015) 68, no. 1, 168–181, 10.1111/prd.12064, 2-s2.0-84927127483. 25867986

[55] Marconi G. D. , Fonticoli L. , and Rajan T. S. , et al.Epithelial-Mesenchymal Transition (EMT): The Type-2 EMT in Wound Healing, Tissue Regeneration and Organ Fibrosis, Cells. (2021) 10, no. 7, 10.3390/cells10071587, 1587. 34201858 PMC8307661

[56] Mai J. , Virtue A. , Shen J. , Wang H. , and Yang X.-F. , An Evolving New Paradigm: Endothelial Cells—Conditional Innate Immune Cells, Journal of Hematology & Oncology. (2013) 6, no. 1, 10.1186/1756-8722-6-61, 2-s2.0-84882329201, 61. 23965413 PMC3765446

[57] Higuchi H. , Kurose I. , and Fukumura D. , et al.Active Oxidants Mediate IFN-Alpha-Induced Microvascular Alterations in Rat Mesentery, The Journal of Immunology. (1997) 158, no. 10, 4893–4900, 10.4049/jimmunol.158.10.4893. 9144506

[58] Li Q. , Zhu Z. , and Wang L. , et al.Single-Cell Transcriptome Profiling Reveals Vascular Endothelial Cell Heterogeneity in Human Skin, Theranostics. (2021) 11, no. 13, 6461–6476, 10.7150/thno.54917. 33995668 PMC8120211

[59] Suárez-López Del Amo F. , Garaicoa-Pazmiño C. , Fretwurst T. , Castilho R. M. , and Squarize C. H. , Dental Implants-Associated Release of Titanium Particles: A Systematic Review, Clinical Oral Implants Research. (2018) 29, no. 11, 1085–1100, 10.1111/clr.13372, 2-s2.0-85054335706. 30280418

[60] Alhamad M. , Ricardo Barao V. A. , Sukotjo C. , Yerokhin A. , and Mathew M. T. , Unpredictable Electrochemical Processes in Ti Dental Implants: The Role of Ti Ions and Inflammatory Products, ACS Applied Bio Materials. (2023) 6, no. 9, 3661–3673, 10.1021/acsabm.3c00235. 37602778

[61] Djinic Krasavcevic A. , Nikolic N. , and Milinkovic I. , et al.Notch Signalling Cascade and Proinflammatory Mediators in Peri-Implant Lesions With Different RANKL/OPG Ratios—An Observational Study, Journal of Periodontal Research. (2023) 58, no. 2, 360–368, 10.1111/jre.13096. 36617525

[62] Milinkovic I. , Djinic Krasavcevic A. , and Nikolic N. , et al.Notch Down-Regulation and Inflammatory Cytokines and RANKL Overexpression Involvement in Peri-Implant Mucositis and Peri-Implantitis: A Cross-Sectional Study, Clinical Oral Implants Research. (2021) 32, no. 12, 1496–1505, 10.1111/clr.13850. 34546593

[63] Zhang Q. , Liu J. , Ma L. , Bai N. , and Xu H. , Wnt5a is Involved in LOX-1 and TLR4 Induced Host Inflammatory Response in Peri-Implantitis, Journal of Periodontal Research. (2020) 55, no. 2, 199–208, 10.1111/jre.12702, 2-s2.0-85073949587. 31593304

