# Supplementary figures and images for: Single‐Cell Transcriptomics Reveal Microenvironment Alterations in Canine Peri‐Implantitis
Source: Mediators Inflamm. 2025 Dec 31;2025:9937505. doi: 10.1155/mi/9937505 (PMC12767392; doi:10.1155/mi/9937505)

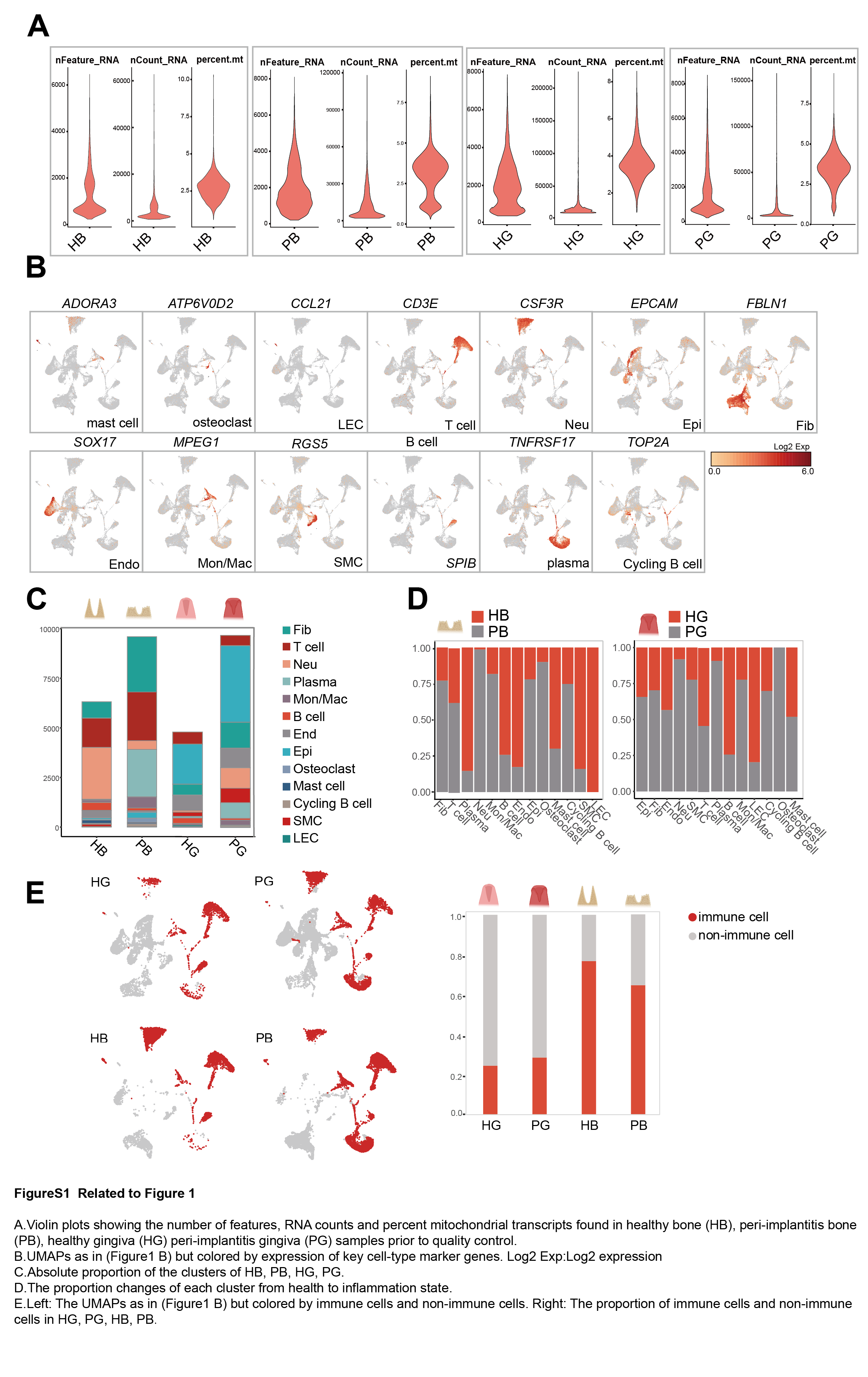

Supplement: Supplementary file 1 — Supporting Information 1 Figure S1 related to Figure 1. (a) Violin plots showing the number of features, RNA counts and percentage of mitochondrial transcripts found in HB, PB, HG, PG samples prior to quality control. (b) UMAPs are the same as those in Figure 1b but are colored according to the expression of key cell‐type markers. Log2 Exp: Log2 expression. (c) Absolute proportions of the clusters of HB, PB, HG, and PG. (c) The proportion changes in each cluster from the healthy state to the inflammatory state. (d) Left: Proportion of each cluster of HB, PB. Right: Proportion of each cluster of HG, PG. (e) Left: The UMAPs as in Figure 1b but colored by immune cells and nonimmune cells. Right: The proportion of immune cells and nonimmune cells in HG, PG, HB, and PB. [file MI-2025-9937505-s001.png]

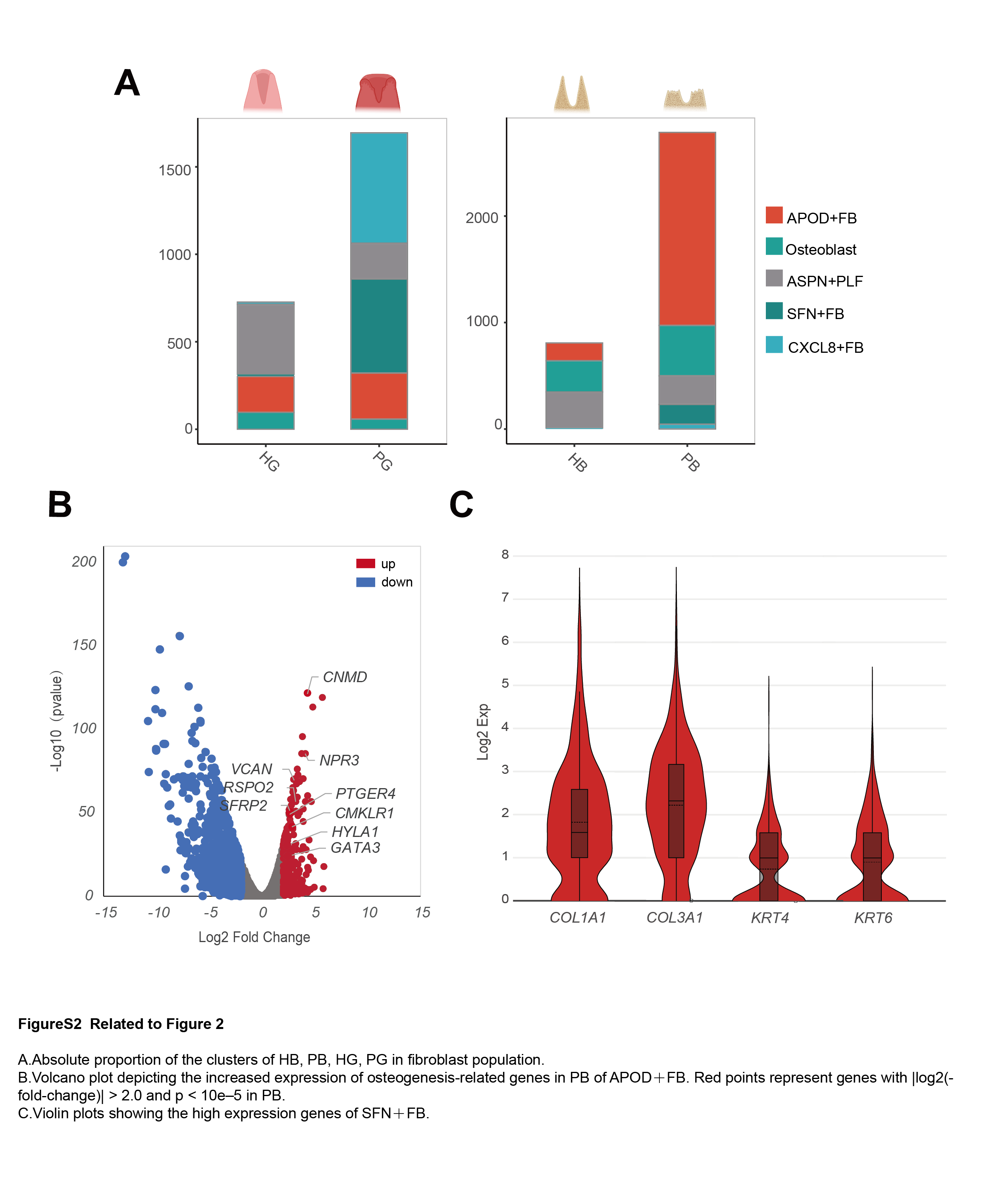

Supplement: Supplementary file 2 — Supporting Information 2 Figure S2 related to Figure 2. (a) Absolute proportions of the clusters of HB, PB, HG, and PG in the fibroblast population. (b) Volcano plot depicting the increased expression of osteogenesis‐related genes in the PB of APOD+Fib. Red points represent genes with FDR < 0.1, log2 (fold change) > 2.0 and adjusted p value < 0.05 in PB. Blue points represent genes with FDR < 0.1, log2 (fold change) < −2.0 and adjusted p value < 0.05 in PB. (c) Violin plot showing the genes highly expressed in SFN+Fib. [file MI-2025-9937505-s002.png]

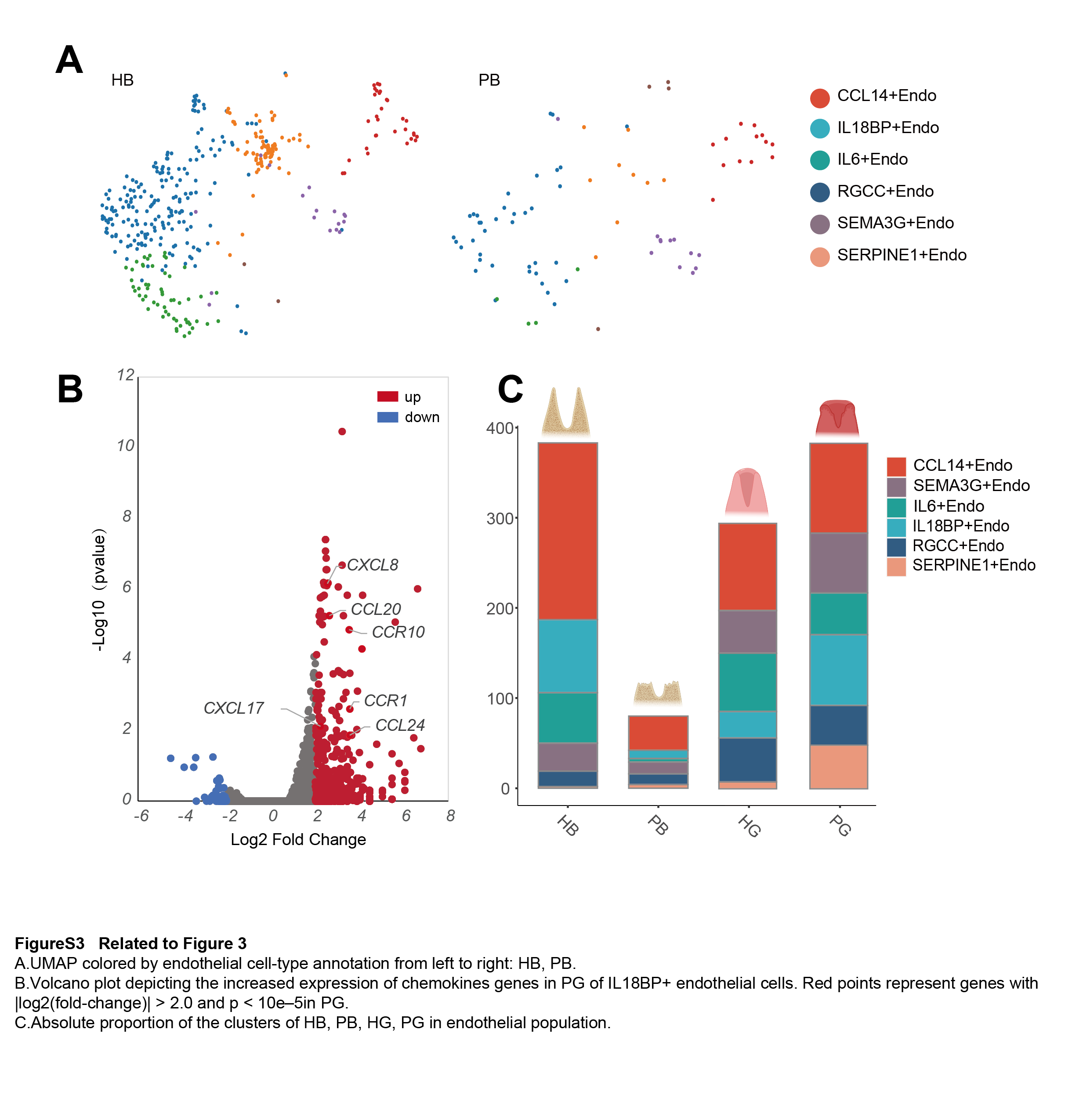

Supplement: Supplementary file 3 — Supporting Information 3 Figure S3 related to Figure 3. (a) UMAP of endothelial cells as in Figure 1a, annotated and colored by the sample type of origin (HB, PB) and subclustering. (b) Volcano plot depicting the increased expression of cytokine genes in the PG of IL18BP+ endothelial cells. Red points represent genes with log2 (fold change) > 2.0 and adjusted p value <0.05 in the PG. (c) Absolute proportion of the clusters of HB, PB, HG, and PG in the endothelial population. [file MI-2025-9937505-s005.png]

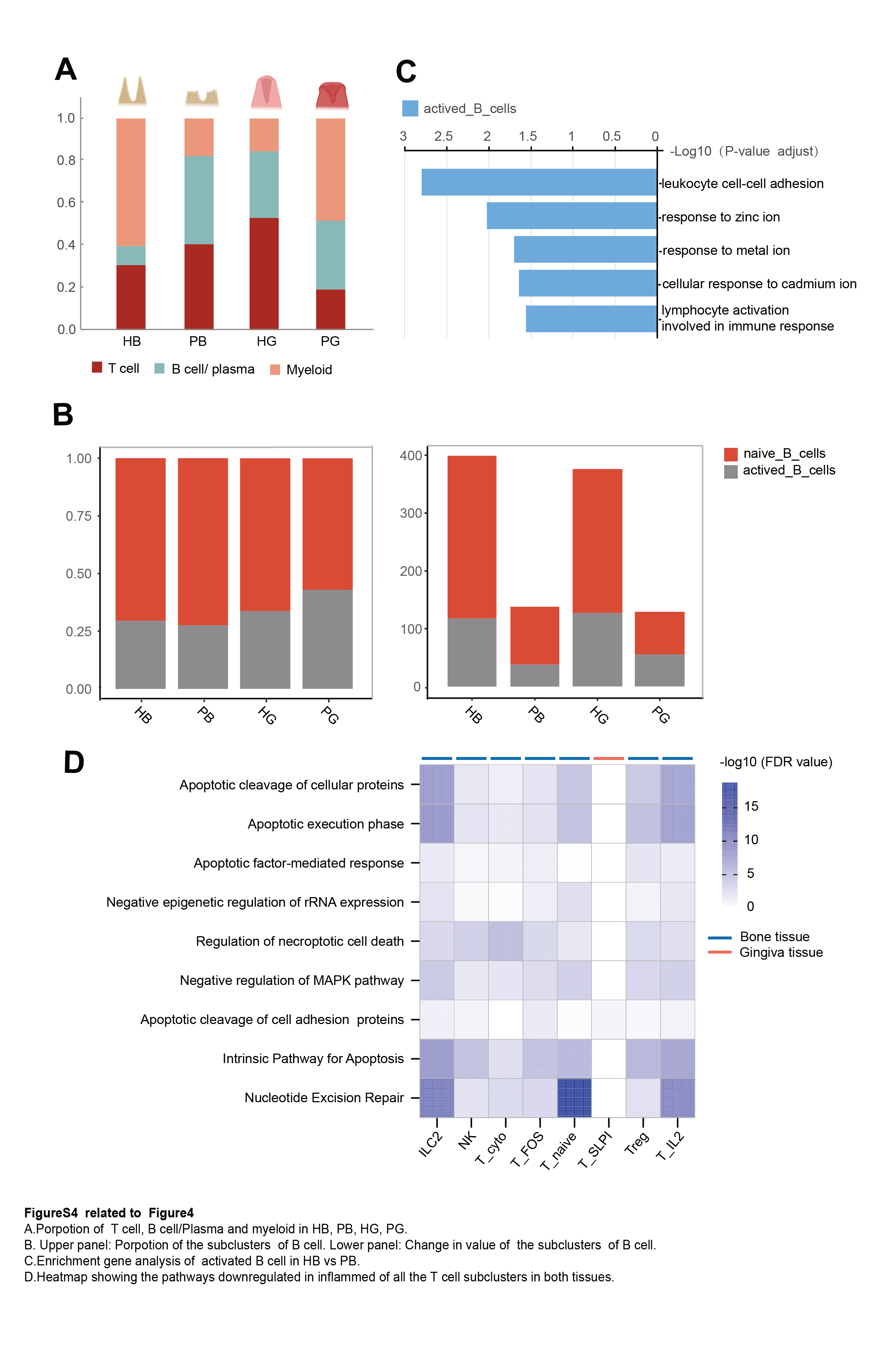

Supplement: Supplementary file 4 — Supporting Information 4 Figure S4 related to Figure 5. (a) Proportion of T cells, B cells/plasma and myeloid cells in the HB, PB, HG, and PG. (b) Upper panel: Proportion of the subclusters of B cells. Lower panel: Absolute proportion of the subclusters of B cells. (c) Enrichment gene analysis of activated B cells in HB versus PB. (d) Heatmap showing the pathways downregulated in all the T cell subclusters in both tissues. [file MI-2025-9937505-s003.png]

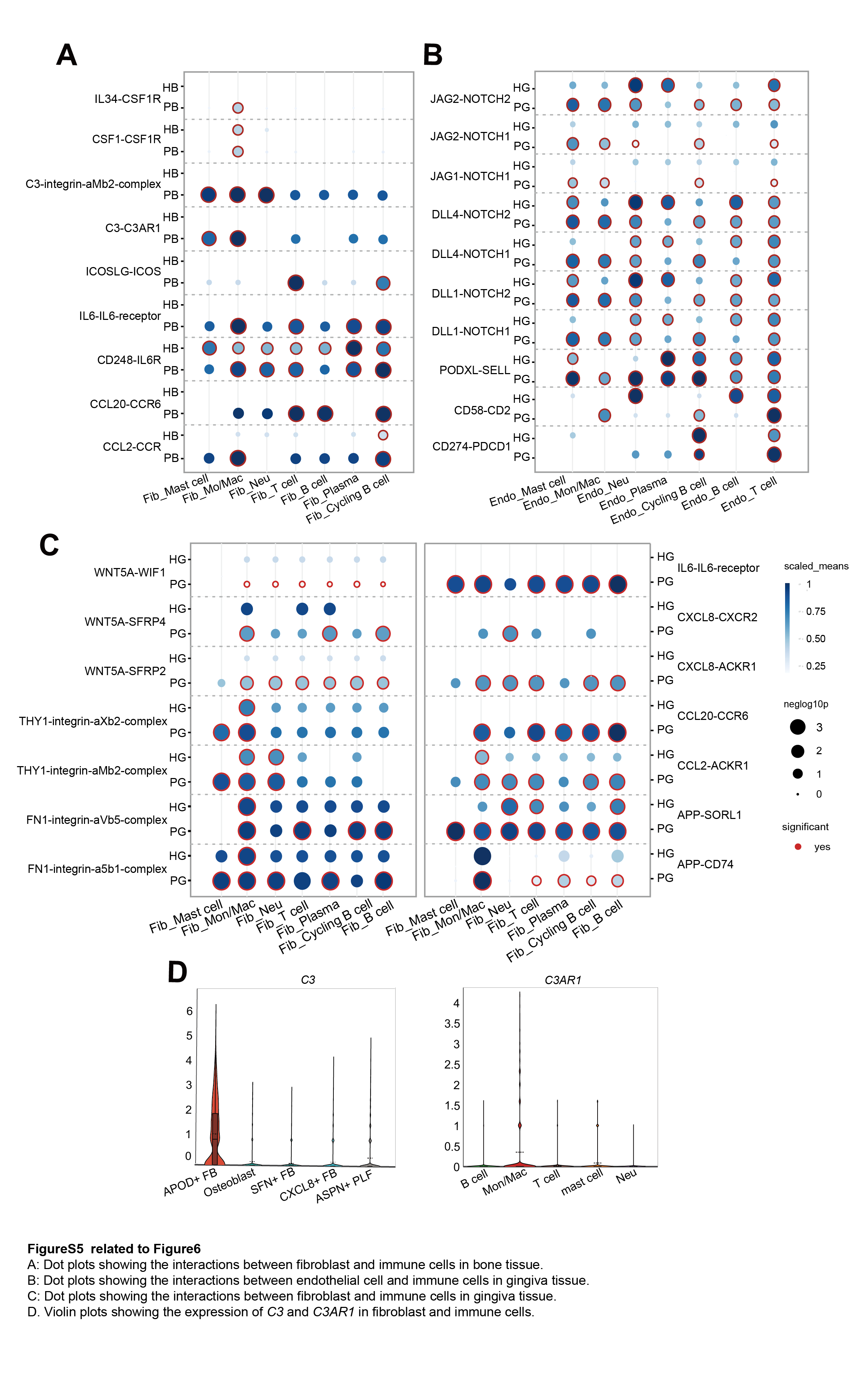

Supplement: Supplementary file 5 — Supporting Information 5 Figure S5 related to Figure 6. (a) Dot plot showing the interactions between fibroblasts and immune cells in bone tissue. (b) Dot plot showing the interactions between endothelial cells and immune cells in gingival tissue. (c) Dot plots showing the interactions between fibroblasts and immune cells in gingival tissue. (d) Violin plots showing the expression of C3 and C3AR1 in fibroblasts and immune cells. [file MI-2025-9937505-s004.png]
